# Naphthalene-Type Glycosides from *Rumex obtusifolius* Roots and Their Protective Effects Against Muscle Atrophy in C2C12 Myotubes

**DOI:** 10.3390/pharmaceutics18070807

**Published:** 2026-06-29

**Authors:** Yun Seok Joh, Jung Eun Park, Moon Jin Ra, Sang Mi Jung, Gabsik Yang, Ki Sung Kang, Ki Hyun Kim

**Affiliations:** 1 School of Pharmacy, Sungkyunkwan University, Suwon 16419, Republic of Korea; ysjoh05@g.skku.edu; 2 College of Korean Medicine, Gachon University, Seongnam 13120, Republic of Korea; ppp1416@gachon.ac.kr (J.E.P.); yanggs@gachon.ac.kr (G.Y.); 3 Hongcheon Institute of Medicinal Herb, Hongcheon-gun 25142, Republic of Korea; ramj90@himh.re.kr (M.J.R.); sgmo77@naver.com (S.M.J.)

**Keywords:** *Rumex obtusifolius*, naphthalene-type glycosides, C2C12, muscle atrophy, dexamethasone

## Abstract

**Background/Objectives:** *Rumex obtusifolius* L. (Polygonaceae) has been traditionally used to treat various disorders, including hepatic and gastrointestinal diseases. However, the phytochemical constituents of its roots and their potential protective effects against skeletal muscle atrophy remain poorly understood. This study aimed to isolate and characterize bioactive constituents from *R. obtusifolius* roots and evaluate their protective effects against dexamethasone (DEX)-induced muscle atrophy in C2C12 myotubes. **Methods:** LC–MS-guided phytochemical investigation of the ethanol extract of *R. obtusifolius* roots, followed by successive column chromatography and HPLC purification, resulted in the isolation of four naphthalene-type glycosides. Their structures were elucidated using 1D and 2D NMR spectroscopy, HR-ESIMS, and chemical transformation. The protective effects of compounds **1** and **4** against dexamethasone (DEX)-induced muscle atrophy were evaluated by assessing myotube morphology, myogenic and atrophy-related protein expression, and PI3K/Akt/mTOR signaling. **Results:** A new naphthalene malonylglucoside, nepodin-8-*O*-*β*-D-(6′-*O*-malonyl)-glucopyranoside (**1**), together with three known glycosides (**2**–**4**), was identified. Among the isolated compounds, compound **1** significantly attenuated DEX-induced muscle atrophy in a concentration-dependent manner by increasing myotube diameter and improving myotube morphology. It restored the expression of the myogenic markers MyoD and myogenin while suppressing the atrophy-related proteins MuRF1 and MAFBX. Furthermore, compound **1** reversed DEX-induced suppression of the PI3K/Akt/mTOR signaling pathway, indicating recovery of anabolic signaling. **Conclusions:** This study reports a new naphthalene malonylglucoside (**1**) from *R. obtusifolius* roots and demonstrates that compound **1** protects against DEX-induced skeletal muscle atrophy through restoration of myogenic differentiation and activation of the PI3K/Akt/mTOR pathway. These findings suggest that compound **1** is a promising natural lead compound for the development of therapeutics targeting muscle wasting disorders.

## 1. Introduction

Skeletal muscle atrophy is a pathological condition characterized by a progressive loss of muscle mass, strength, and function, leading to impaired mobility and an increased risk of metabolic and chronic diseases [1,2]. It is commonly associated with aging (sarcopenia), prolonged glucocorticoid exposure, physical inactivity, and chronic illnesses. At the molecular level, muscle atrophy is primarily driven by an imbalance between protein synthesis and degradation, in which the ubiquitin–proteasome system plays a central role through the upregulation of key E3 ubiquitin ligases, such as muscle RING finger 1 (MuRF1) and muscle atrophy F-box (MAFBX/Atrogin-1) [3]. In parallel, the suppression of myogenic regulatory factors, including MyoD and myogenin, contributes to impaired muscle regeneration [4]. Despite the increasing prevalence of muscle atrophy-related conditions, effective therapeutic strategies remain limited, highlighting the need to identify novel bioactive compounds with anti-atrophic potential.

Natural products have long been recognized as a valuable source of structurally diverse and biologically active compounds, offering promising opportunities for the discovery of new therapeutic agents [5]. In particular, plant-derived secondary metabolites have demonstrated a wide range of pharmacological activities, including anti-inflammatory, antioxidant, and metabolic regulatory effects, which are closely associated with the pathogenesis of muscle atrophy [6].

*Rumex obtusifolius* L. (broad-leaved dock) is a perennial herbaceous plant belonging to the family Polygonaceae and is widely distributed across temperate regions of Europe and Asia [7]. The species is morphologically characterized by large ovate leaves with undulating margins, reddish stems and veins, and inconspicuous green flowers arranged along elongated inflorescences [7,8]. The leaves typically form a basal rosette, and the fruit consists of distinctive triangular, winged achenes [7,9]. Traditionally, *R. obtusifolius* has been used in various systems of folk medicine to treat a broad range of ailments, including dermatological conditions, gastrointestinal disorders, and hepatic diseases [10,11]. The roots, in particular, have been widely utilized to support liver function and to manage symptoms such as fever and jaundice [12]. In addition, topical applications of root and leaf extracts have been employed for the treatment of wounds, abscesses, and inflammatory skin conditions [9,11]. These traditional uses suggest potential pharmacological relevance, particularly in the context of inflammation, oxidative stress, and liver-associated disorders, which remain major targets in contemporary drug discovery.

*R. obtusifolius* is known for its strong adaptability to disturbed environments, including agricultural fields and roadside habitats, where it exhibits competitive growth behavior. This ecological resilience is often associated with the production of bioactive secondary metabolites that contribute to plant defense and environmental interactions. Phytochemical investigations of *Rumex* species have revealed the presence of diverse bioactive secondary metabolites, including anthraquinones (e.g., emodin and chrysophanol), flavonoids, and naphthalene derivatives [8,13,14]. Although anthraquinones and flavonoids from *Rumex* species have been extensively studied, the chemical diversity of minor constituents, particularly glycosylated and structurally unique metabolites, remains insufficiently explored. In recent years, increasing attention has been directed toward the structural diversity and pharmacological potential of naphthalene glycosides isolated from *Rumex* species [15]. These compounds exhibit a wide range of biological activities, including antimicrobial, antioxidant, anti-inflammatory, and hepatoprotective effects [11,12,13]. However, despite reports on diverse biological activities, the underlying molecular mechanisms and specific biological targets of these compounds remain largely unclear. Recent studies have demonstrated that oxidative stress and inflammation are major contributors to skeletal muscle atrophy and muscle protein degradation. Given the reported antioxidant and anti-inflammatory activities of naphthalene glycosides from *Rumex* species [11,12,13], these compounds may represent promising candidates for the modulation of muscle atrophy-related pathways. However, their potential effects on skeletal muscle atrophy have not yet been investigated.

As part of our continuing efforts to discover novel bioactive natural products from natural sources [16,17,18,19,20], we investigated the ethanol extract of *R. obtusifolius* roots to identify and characterize new secondary metabolites. Using a combination of repeated column chromatography and high-performance liquid chromatography (HPLC), followed by structural analysis using liquid chromatography–mass spectrometry (LC–MS), we isolated four naphthalene-type glycosides from the root extract of *R. obtusifolius*. Among these, compound **1** was identified as a new naphthalene malonylglucoside. Its structure was elucidated based on comprehensive spectroscopic analyses, including one- and two-dimensional nuclear magnetic resonance (1D and 2D NMR; ^1^H–^1^H COSY, HSQC, and HMBC) and high-resolution electrospray ionization mass spectrometry (HR-ESIMS), in combination with chemical reaction. Herein, we report the isolation and structural characterization of these naphthalene-type glycosides and evaluate their protective effects against DEX-induced muscle atrophy in C2C12 myotubes, along with an investigation of the underlying molecular mechanisms.

## 2. Materials and Methods

### 2.1. General Experimental Procedures

Optical rotations were measured using a Jasco P-2000 polarimeter (Jasco, Easton, MD, USA). Ultraviolet (UV) spectra were recorded on an Agilent 8453 UV–visible spectrophotometer (Agilent Technologies, Santa Clara, CA, USA). Nuclear magnetic resonance (NMR) spectra were recorded on a Bruker AVANCE III HD 850 MHz NMR spectrometer (Bruker, Karlsruhe, Germany), operating at 850 MHz for ^1^H and 212.5 MHz for ^13^C. Chemical shifts are reported in parts per million (ppm, δ). LC–MS analyses were performed on an Agilent 1200 Series HPLC system equipped with a diode array detector and a 6130 Series electrospray ionization (ESI) mass spectrometer, using an analytical Kinetex C18 100 Å column (100 × 2.1 mm, 5 μm; flow rate, 0.3 mL/min; Phenomenex, Torrance, CA, USA). High-resolution electrospray ionization mass spectrometry (HR-ESIMS) data were acquired using an Agilent 6545 Q-TOF LC/MS system (Agilent Technologies, Santa Clara, CA, USA). Semi-preparative HPLC was carried out using a Waters 2695 binary HPLC pump equipped with a Waters 2998 photodiode array detector (Waters Corporation, Milford, CT, USA), fitted with a Phenomenex Luna C18 column (250 mm × 10 mm, 10 μm; flow rate, 5 mL/min; Phenomenex, Torrance, CA, USA). Silica gel 60 (230–400 mesh; Merck, Darmstadt, Germany) and RP-C18 silica gel (230–400 mesh; Merck) were used for column chromatography. Precoated silica gel F254 and RP-C18 F254s plates (Merck) were used for thin-layer chromatography (TLC). TLC spots were visualized under UV light or by heating after spraying with anisaldehyde–sulfuric acid reagent.

### 2.2. Plant Material

The roots of *Rumex obtusifolius* L. were collected in Eumseong-gun, Chungcheongbuk-do, Republic of Korea, in May 2024. The plant material was identified by one of authors, Ki Hyun Kim at School of Pharmacy, Sungkyunkwan University, Republic of Korea. A voucher specimen (HIMH-2404) has been deposited in the herbarium of the Nakdonggang National Institute of Biological Resources, Sangju, Republic of Korea.

### 2.3. Extraction and Isolation

Completely dried roots of *R. obtusifolius* L. (500 g) were extracted three times (5.0 L × 3) with 80% ethanol (EtOH) by sonication at room temperature, followed by filtration. The combined filtrates were concentrated under reduced pressure to yield a crude EtOH extract (43.4 g). The extract was suspended in distilled water (700 mL) and successively partitioned with *n*-hexane, dichloromethane (CH_2_Cl_2_), ethyl acetate (EtOAc), and *n*-butanol (*n*-BuOH) (700 mL × 3 for each solvent), affording 3.5 g, 0.4 g, 4.4 g, and 6.3 g of each fraction, respectively. LC–MS analysis indicated that the EtOAc-soluble fraction contained the interesting compounds. The EtOAc fraction (4.4 g) was subjected to silica gel column chromatography using a gradient solvent system of CH_2_Cl_2_–MeOH (50:1 to 1:1, *v*/*v*) to yield five subfractions (E1–E5). Subfraction E5 (1.5 g) was further separated by reversed-phase column chromatography using a CH_3_OH–H_2_O gradient system (6:4 to 8:2, *v*/*v*), yielding five subfractions (E5_1–E5_5). Subfraction E5_3 (280 mg) was purified by semi-preparative reversed-phase HPLC (Phenomenex Luna C18, 250 mm × 10 mm i.d., 5 μm) using an isocratic system of 27% acetonitrile (CH_3_CN)/H_2_O (flow rate, 2 mL/min) to afford compounds **1** (10.6 mg, *t*_R_ = 48.5 min), **2** (0.7 mg, *t*_R_ = 59.5 min), **3** (0.8 mg, *t*_R_ = 28.0 min), and **4** (8.1 mg, *t*_R_ = 34.5 min).

#### 2.3.1. Nepodin-8-*O*-*β*-d-(6′-O-Malonyl)-Glucopyranoside (**1**)

White amorphous powder; ${\left[\alpha \right]}_{D}^{20}$ −55.0 (*c* 0.09, CH_3_CN); UV (CH_3_OH) *λ*_max_ (log ε) 224 (3.9) nm; ^1^H (DMSO-*d*_6_, 850 MHz) and ^13^C NMR (DMSO-*d*_6_, 212.5 MHz) see Table 1; HR-ESIMS (positive ion mode) *m*/*z*: 465.1394 [M + H]^+^ (calcd. For C_22_H_25_O_11_, 465.1397) (Figures S1–S6).

#### 2.3.2. Torachrysone-8-*O*-*β*-D-(6′-O-Malonyl)-Glucopyranoside (**2**)

White amorphous powder; ${\left[\alpha \right]}_{D}^{20}$ −48.0 (*c* 0.04, CH_3_CN); ^1^H (DMSO-*d*_6_, 850 MHz) and ^13^C NMR (DMSO-*d*_6_, 212.5 MHz) see Table 1; HR-ESIMS (positive ion mode) *m*/*z*: 495.1502 [M + H]^+^ (calcd. For C_23_H_27_O, 495.1503) (Figures S7–S10).

#### 2.3.3. Nepodin-8-*O*-*β*-d-Glucopyranoside (**3**)

White amorphous powder; ${\left[\alpha \right]}_{D}^{20}$ +61.5 (*c* 0.04, MeOH); ^1^H NMR (CD_3_OD, 850 MHz): *δ*_H_ 7.32 (1H, d, *J* = 8.0 Hz, H-5), 7.27 (1H, t, *J* = 8.0 Hz, H-6), 7.23 (1H, d, *J* = 8.0 Hz, H-7), 7.05 (1H, s, H-4), 5.02 (1H, d, *J* = 8.0 Hz, H-1′), 3.47 (1H, m, H-2′), 3.43 (1H, m, H-3′), 3.40 (1H, m, H-4′), 3.34 (1H, m, H-5′), 3.65 (1H, dd, *J* = 12.0, 6.0 Hz, H-6′a), 3.86 (1H, dd, *J* = 12.0, 2.0 Hz, H-6′b), 2.50 (3H, s, H-12), 2.21 (3H, d, *J* = 0.5 Hz, H-13); HR-ESIMS (positive ion mode) *m*/*z*: 379.1391 [M + H]^+^ (calcd. For C_19_H_23_O_8_, 379.1387) (Figures S11–S13).

#### 2.3.4. Torachrysone-8-*O*-*β*-d-Glucopyranoside (**4**)

White amorphous powder; ${\left[\alpha \right]}_{D}^{20}$ +52.3 (*c* 0.04, MeOH); ^1^H NMR (acetone-*d*_6_, 850 MHz): *δ*_H_ 6.95 (1H, s, H-4), 6.92 (1H, d, *J* = 2.0 Hz, H-5), 6.77 (1H, d, *J* = 2.0 Hz, H-7), 5.10 (1H, d, *J* = 7.5 Hz, H-1′), 3.77 (3H, s, OCH_3_), 3.56 (1H, m, H-2′), 3.53 (1H, m, H-3′), 3.50 (1H, m, H-4′), 3.40 (1H, m, H-5′), 3.65 (1H, dd, *J* = 12.0, 6.0 Hz, H-6′a), 3.86 (1H, dd, *J* = 12.0, 2.5 Hz, H-6′b), 2.41 (3H, s, H-12), 2.17 (3H, d, *J* = 1.0 Hz, H-13); HR-ESIMS (positive ion mode) *m*/*z*: 409.1494 [M + H]^+^ (calcd. For C_20_H_25_O_9_, 409.1493) (Figures S14–S16).

### 2.4. Acid Hydrolysis and Absolute Configuration Determination of Sugar Moieties

Compound **1** (1.5 mg) was hydrolyzed with 1 N HCl at 80 °C for 2 h, followed by extraction with EtOAc. The aqueous layer was neutralized by repeated evaporation under reduced pressure and then dissolved in anhydrous pyridine (0.5 mL) containing L-cysteine methyl ester hydrochloride (1.0 mg). The reaction mixture was heated at 60 °C for 1 h, after which *o*-tolyl isothiocyanate (50 μL) was added, and the mixture was further heated at 60 °C for an additional 1 h. The reaction mixture was evaporated under reduced pressure and dissolved in MeOH. The resulting solution was directly analyzed by LC–MS using a gradient system of MeOH/H_2_O (1:9 → 7:3, 0–30 min; 100% MeOH, 31–41 min; 0% MeOH, 42–52 min) at a flow rate of 0.3 mL/min, on an analytical Kinetex C_18_ 100 Å column (100 mm × 2.1 mm i.d., 5 μm). The sugar moiety of compound **1** was identified as D-glucopyranose by comparison of its retention time with that of an authentic standard.

### 2.5. Cell Culture

C2C12 myoblasts were cultured in DMEM supplemented with 10% FBS at 37 °C in a 5% CO_2_ atmosphere. To induce differentiation, once the cells reached 80% confluence, the medium was replaced with differentiation medium consisting of DMEM supplemented with 2% horse serum. The medium was changed every 2 days, and differentiation was allowed to proceed for 7 days until multinucleated myotubes were formed. To induce muscle atrophy, differentiated C2C12 myotubes were treated with dexamethasone (DEX) at 10 μM. Compounds **1** and **4** were administered at the indicated concentrations simultaneously with DEX treatment.

### 2.6. Cell Viability Assay

Cell viability was assessed using an EZ-Cytox Cell Viability Assay Kit (DoGenBio, Seoul, Republic of Korea) according to the manufacturer’s instructions. C2C12 cells were seeded in 96-well plates and cultured for 24 h prior to treatment. The cells were then treated with compounds **1** and **4** at concentrations ranging from 0 to 400 μM for 24 h. Following treatment, EZ-Cytox reagent was added to each well, and the plates were incubated for 1 h at 37 °C. Absorbance was measured at 450/650 nm using a microplate reader, and cell viability was expressed as a percentage relative to the untreated control group.

### 2.7. Hematoxylin and Eosin (H&E) Staining

C2C12 cells were seeded in 6-well plates at a density of 2 × 10^5^ cells per well and differentiated under appropriate conditions. After treatment with samples or vehicle control, the cells were washed with PBS and fixed with 4% paraformaldehyde for 15 min at room temperature. Following fixation, the cells were washed with PBS and stained with hematoxylin for 5 min. After rinsing with running tap water, the cells were counterstained with eosin for 2 min. Myotube diameter was measured from nine myotubes per group and expressed as a percentage of the untreated control (N.T.) group, which was defined as 100%.

### 2.8. Western Blot Analysis

C2C12 cells were lysed using RIPA buffer containing protease and phosphatase inhibitors. The protein concentration was determined using a bicinchoninic acid assay. Equal amounts of protein were separated by SDS-PAGE and transferred onto polyvinylidene difluoride (PVDF) membranes. The membranes were blocked with 5% skim milk in Tris-buffered saline containing 0.1% Tween-20 (TBST) for 1 h at room temperature and incubated overnight at 4 °C with primary antibodies against MyoD, myogenin, MAFBX/Atrogin-1, MuRF1, p-PI3K, PI3K, p-Akt, Akt, p-mTOR, and mTOR (Cell Signaling Technology, Danvers, MA, USA). After washing with TBST, the membranes were incubated with horseradish peroxidase-conjugated secondary antibodies for 1 h at room temperature. Protein bands were visualized using an enhanced chemiluminescence detection system and captured using an imaging system. Band intensities were quantified using ImageJ software (version 1.54; National Institutes of Health, Bethesda, MD, USA). MyoD, myogenin, MAFBX/Atrogin-1, and MuRF1 expression levels were normalized to glyceraldehyde 3-phosphate dehydrogenase (GAPDH), whereas phosphorylated PI3K, Akt, and mTOR levels were normalized to their respective total protein levels.

### 2.9. Statistical Analysis

All experiments were performed in triplicate (*n* = 3), and data are presented as mean ± SEM. Statistical significance among groups was determined using one-way analysis of variance (ANOVA) followed by Tukey’s post hoc test using GraphPad Prism software (version 11.0.0, GraphPad Software, San Diego, CA, USA). A value of *p* < 0.05 was considered statistically significant.

## 3. Results

### 3.1. Isolation and Structural Characterization of Naphthalene-Type Glycosides

**Figure 1:**
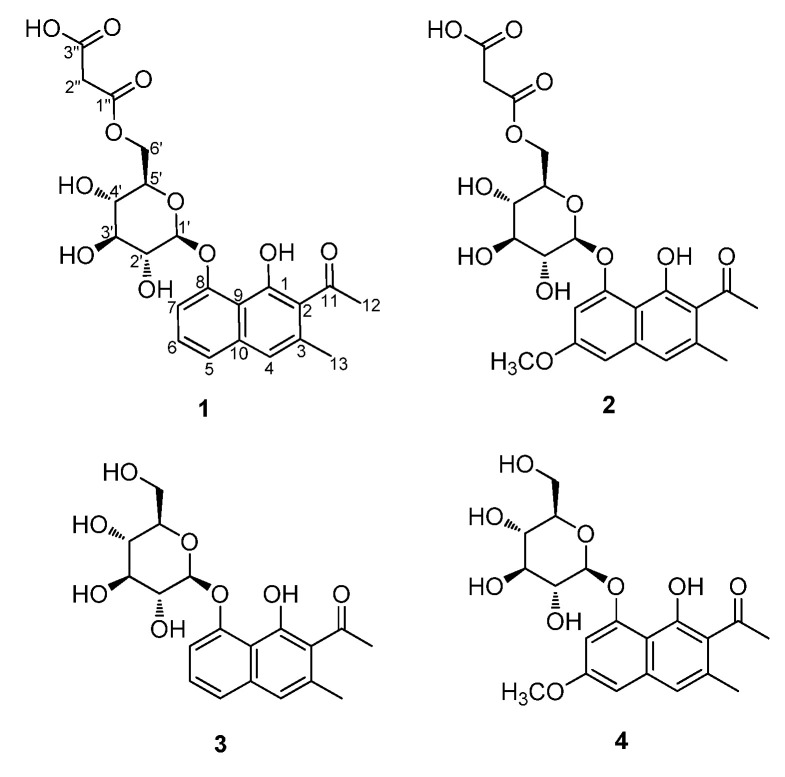
Chemical structures of compounds **1**–**4**.

To discover new bioactive compounds, a phytochemical investigation was conducted on the EtOH extract of the roots of *R. obtusifolius*. The crude extract was subjected to successive solvent partitioning with four organic solvents using *n*-hexane, CH_2_Cl_2_, EtOAc, and *n*-BuOH to yield the corresponding soluble fractions. Among these, LC–MS analysis indicated that the EtOAc-soluble fraction contained naphthalene-type glycosides. Guided by LC–MS data, this fraction was further purified using a combination of chromatographic techniques, followed by semi-preparative HPLC. Through this process, four naphthalene-type glycosides were successfully isolated (Figure 1).

**Figure 2:**
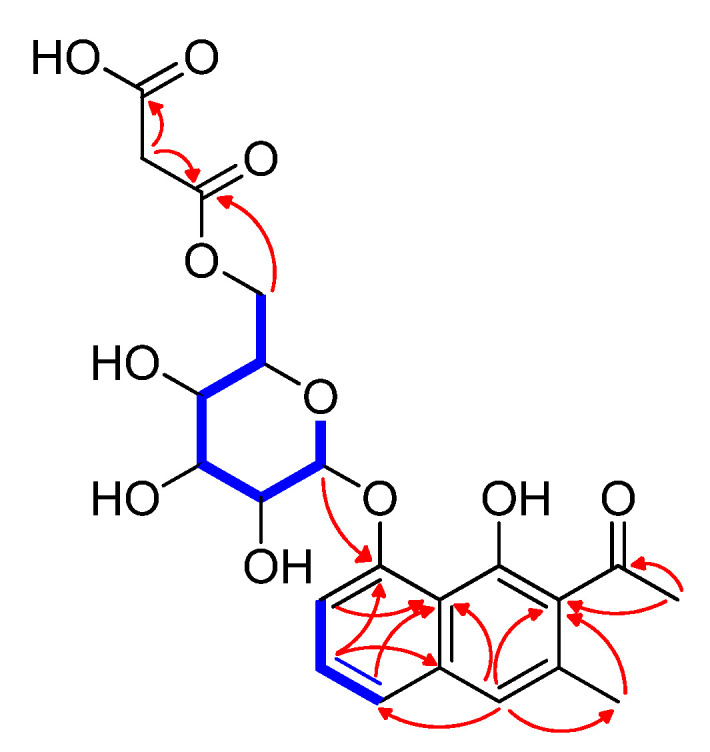
Key COSY (blue bold) and HMBC (red arrow) correlations for compound **1**.

Compound **1** was isolated as a white amorphous powder, and the molecular formula of C_22_H_24_O_11_ was determined from the HRESIMS peak at *m*/*z* 465.1394 [M + H]^+^ (calcd. For C_22_H_25_O_11_, 465.1397) and the NMR data (Table 1). The ^1^H NMR spectrum of compound **1** indicated the presence of two methyl resonances at *δ*_H_ 2.53 (3H, s) and 2.25 (3H, s), a methylene signal at *δ*_H_ 3.42 (2H, m), and four aromatic proton signals at *δ*_H_ 7.48 (1H, d, *J* = 8.0 Hz), 7.44 (1H, t, *J* = 8.0 Hz), 7.26 (1H, d, *J* = 8.0 Hz), and 7.22 (1H, s), in addition to signals corresponding to the glucose moiety, including an anomeric proton at *δ*_H_ 5.09 (1H, d, *J* = 8.0 Hz). The ^13^C NMR and HSQC spectra revealed 22 carbon signals, including two methyl carbons (*δ*_C_ 32.0 and 19.5), four aromatic methine carbons (*δ*_C_ 127.4, 122.5, 119.3, and 110.6), three carbonyl carbons (*δ*_C_ 205.0, 168.2, and 167.6), a glucopyranosyl unit (*δ*_C_ 102.2, 75.1, 74.2, 73.1, 69.8, and 63.6), and one methylene carbon (*δ*_C_ 41.7). The ^1^H and ^13^C NMR spectra of compound **1** (Table 1) displayed characteristic signals for a naphthalene-type glycoside. The NMR data were similar to those of compound **2**, with notable differences, including the absence of a methoxy group signal at *δ*_H_ 3.83 (3H, s) and variations in the aromatic substitution pattern. Furthermore, the NMR spectra of compound **1** closely resembled those of compound **3**, with a key difference being the presence of signals corresponding to a malonyl unit at *δ*_H_ 3.42 (2H, m) and *δ*_C_ 168.2, 167.6, and 41.7. The proton chemical shifts of H-6′ (*δ*_H_ 4.16 and 4.48) in compound **1** were shifted downfield compared to those of compound **3**, suggesting that the malonyl moiety is attached at C-6′. The position of the malonyl group was further confirmed by an HMBC correlation between H-6′ and C-1″ (Figure 2). The planar structure of compound **1** was established through detailed analysis of 2D NMR data, including ^1^H–^1^H COSY and HMBC correlations (Figure 2).

The absolute configuration of the sugar moiety was determined using an LC–MS/UV-based method [21]. Acid hydrolysis of compound **1** yielded a glucopyranose unit, which was identified as D-glucopyranose by comparison of the retention time of its thiocarbamoyl-thiazolidine derivative with that of an authentic standard using LC–MS analysis. The large coupling constant (*J* = 8.0 Hz) of the anomeric proton indicated a *β*-configuration for the glucopyranose unit [21], thereby confirming the sugar moiety as *β*-D-glucopyranose. Accordingly, the structure of compound **1** was elucidated as nepodin-8-*O*-*β*-d-(6′-*O*-malonyl)-glucopyranoside.

The known compounds were identified as torachrysone-8-*O*-*β*-D-(6′-*O*-malonyl)-glucopyranoside (**2**) [22], nepodin-8-*O*-*β*-D-glucopyranoside (**3**) [23], and torachrysone-8-*O*-*β*-D-glucopyranoside (**4**) [24] by comparison of their NMR spectroscopic data with previously reported values, supported by LC–MS analysis.

### 3.2. Effect of Compounds ***1*** and ***4*** on C2C12 Cell Viability

**Figure 3:**
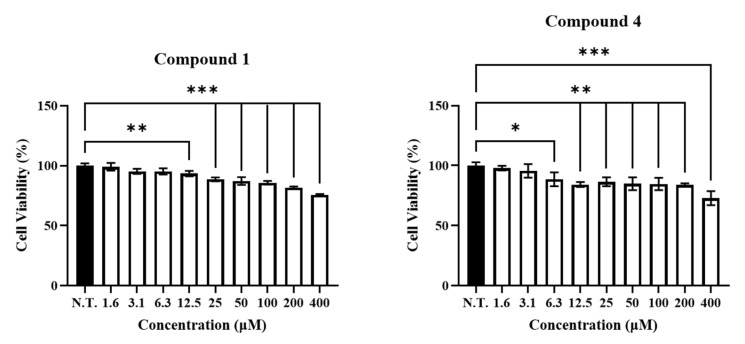
Effects of compounds **1** and **4** on C2C12 cell viability. C2C12 cells were treated with compounds **1** and **4** at the indicated concentrations, and cell viability was measured. Data are presented as the mean ± SD (n ≥ 3). * *p* < 0.05, ** *p* < 0.01, *** *p* < 0.001 vs. untreated control (N.T.) group.

Due to the limited amounts of compounds **2** and **3** obtained during isolation, further biological evaluations were performed only for compounds **1** and **4**. Before investigating the potential effects of compounds **1** and **4** on the regulation of muscle atrophy, we first assessed their effects on C2C12 cell viability. As shown in Figure 3, treatment with compounds **1** and **4** did not significantly affect cell viability at lower concentrations. Compound **1** maintained cell viability at approximately 90% up to 12.5 μM, while a gradual decrease was observed at higher concentrations (25–400 μM), indicating a concentration-dependent reduction. However, cell viability remained above 75% even at the highest concentration tested. Similarly, compound **4** showed no significant reduction in cell viability up to 6.3 μM. A modest but statistically significant decrease was observed from 12.5 μM, with a further gradual decline at higher concentrations (25–400 μM), reaching 72% at 400 μM. Based on these findings, subsequent experiments were performed using non-cytotoxic concentrations of the test compounds. While higher concentrations of compounds **1** and **4** reduced cell viability in a concentration-dependent manner, compound **1** and compound **4** were evaluated at concentrations up to 10 μM and 5 μM, respectively, where no significant cytotoxic effects were observed.

### 3.3. Effects of Compounds ***1*** and ***4*** on Muscle Atrophy in C2C12 Myotubes

**Figure 4:**
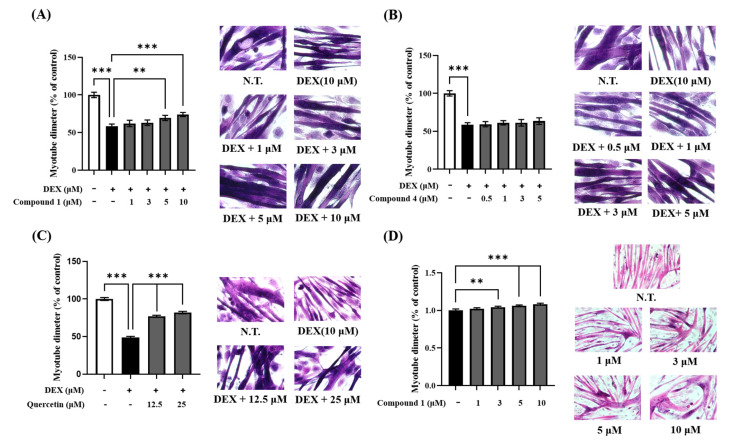
Effects of compounds **1** and **4**, and quercetin on C2C12 myotube morphology under DEX-induced or basal conditions. Differentiated C2C12 myotubes were treated with DEX in the presence or absence of compound **1** (**A**), compound **4** (**B**), or quercetin (**C**). In addition, C2C12 myotubes were treated with compound **1** alone in the absence of DEX to evaluate its direct effect under basal conditions (**D**). Representative H&E images and quantitative analysis of myotube diameter are shown (untreated control, N.T.). Myotube diameter was measured from nine myotubes per group and expressed as a percentage of the untreated control (N.T.) group, which was defined as 100%. Data are expressed as mean ± SD (n ≥ 3). Statistical significance is indicated by brackets. ** *p* < 0.01, *** *p* < 0.001 vs. DEX group.

To evaluate morphological changes associated with muscle atrophy, H&E staining was performed in differentiated C2C12 myotubes (Figure 4). The untreated control (N.T.) group exhibited well-organized, elongated, and multinucleated myotubes, indicating normal myogenic differentiation. In contrast, dexamethasone (DEX)-treated cells showed marked atrophic features, including reduced myotube diameter, disrupted alignment, and decreased myotube density. Treatment with compound **1** significantly attenuated DEX-induced morphological alterations in a concentration-dependent manner. Quantitative analysis revealed that myotube diameter was reduced to 37.5% in the DEX group compared to the untreated control group. Treatment with compound **1** increased myotube diameter by approximately 2.2%, 8.9%, 17.8%, and 15.6% at 1, 3, 5, and 10 μM, respectively, relative to the DEX-treated group. In contrast, treatment with compound **4** showed only marginal and inconsistent changes in myotube diameter, with no statistically significant improvement compared to the DEX group. These results suggest that compound **1** effectively alleviates DEX-induced muscle atrophy in a concentration-dependent manner, whereas compound **4** exhibits a limited effect under the tested conditions. To provide a reference for comparison of the protective effect of compound **1**, quercetin was evaluated in the DEX-induced C2C12 myotube model. DEX treatment markedly reduced myotube diameter compared with the untreated control group, whereas quercetin treatment significantly restored myotube diameter at both 12.5 and 25 μM (Figure 4). Notably, the protective effect of compound **1** at 10 μM was comparable to that observed with quercetin, further supporting its potential as a bioactive constituent against muscle atrophy. In addition, to determine whether compound **1** directly affects myotube morphology under non-atrophic conditions, differentiated C2C12 myotubes were treated with compound **1** in the absence of DEX. Compound **1** alone slightly increased myotube diameter compared with the N.T. group, suggesting that compound **1** may possess anabolic activity that contributes to the maintenance of myotube integrity under basal conditions.

### 3.4. Effects of Compound ***1*** on Protein Expression in C2C12 Myotubes

**Figure 5:**
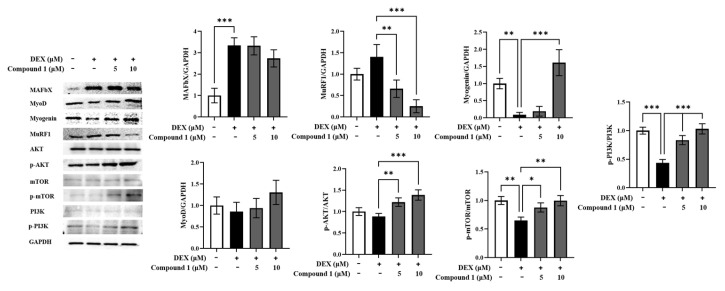
Effects of compound **1** on the expression of myogenic and atrophy-related proteins in DEX-induced C2C12 myotubes. Representative blot images and corresponding quantitative analysis are shown (untreated control, N.T.). Data are expressed as mean ± SD (n ≥ 3). * *p* < 0.05, ** *p* < 0.01, *** *p* < 0.001 vs. N.T. group.

As shown in Figure 5, DEX treatment significantly altered the expression of myogenic and atrophy-related proteins in C2C12 myotubes. The expression levels of MyoD and myogenin were reduced to approximately 86% and 9% of the control level, respectively, whereas MuRF1 expression was increased by approximately 40% compared to the untreated control group. In addition, MAFBX/Atrogin-1 expression was markedly elevated following DEX treatment. Treatment with compound **1** reversed these DEX-induced alterations in a concentration-dependent manner. Specifically, MyoD expression increased by approximately 9.3% and 51.2% at 5 and 10 μM, respectively, compared to the DEX-treated group. Similarly, myogenin expression showed a marked recovery, reaching levels comparable to or higher than those of the untreated control at 10 μM. In contrast, the elevated expression of MuRF1 induced by DEX was significantly suppressed by compound **1**, with reductions of approximately 52.9% and 82.1% at 5 and 10 μM, respectively. Likewise, MAFBX/Atrogin-1 expression was only slightly reduced following compound **1** treatment, decreasing by 0.6% and 18.3% at 5 and 10 μM, respectively, compared with the DEX-treated group. In contrast, compound **1** exerted a more pronounced inhibitory effect on MuRF1 expression than on MAFBX/Atrogin-1 expression.

To further investigate the signaling mechanism underlying the protective effect of compound **1**, the PI3K/Akt/mTOR signaling pathway was additionally examined. DEX treatment markedly decreased the phosphorylation levels of PI3K, Akt, and mTOR compared with those in the untreated control group, indicating suppression of anabolic signaling in C2C12 myotubes. Treatment with compound **1** restored the phosphorylation levels of these signaling proteins in a concentration-dependent manner. In particular, compound **1** at 10 μM significantly increased the p-PI3K/PI3K, p-Akt/Akt, and p-mTOR/mTOR ratios compared with the DEX-treated group. These findings suggest that compound **1** may alleviate DEX-induced muscle atrophy not only by suppressing the expression of atrophy-related proteins, including MuRF1 and MAFBX, but also by restoring PI3K/Akt/mTOR-mediated anabolic signaling in C2C12 myotubes.

## 4. Discussion

Skeletal muscle atrophy is characterized by an imbalance between myogenic regulation and protein degradation, involving myogenic regulatory factors such as MyoD and myogenin, and atrophy-related proteins including MuRF1 and MAFBX/Atrogin-1. In the present study, we demonstrated that naphthalene-type glycosides isolated from *R. obtusifolius* roots, particularly compound **1**, exert protective effects against dexamethasone (DEX)-induced muscle atrophy in C2C12 myotubes.

Dexamethasone (DEX) is widely used to induce muscle atrophy in vitro through the activation of catabolic signaling pathways, including the upregulation of ubiquitin–proteasome system components such as MuRF1 and MAFBX/Atrogin-1, along with the suppression of myogenic regulatory factors [25]. Consistent with previous reports, DEX treatment in this study resulted in reduced myotube diameter and downregulation of MyoD and myogenin, accompanied by increased expression of MuRF1 and MAFBX/Atrogin-1. These changes reflect enhanced protein degradation and impaired myogenic differentiation, ultimately leading to muscle atrophy.

**Figure 6:**
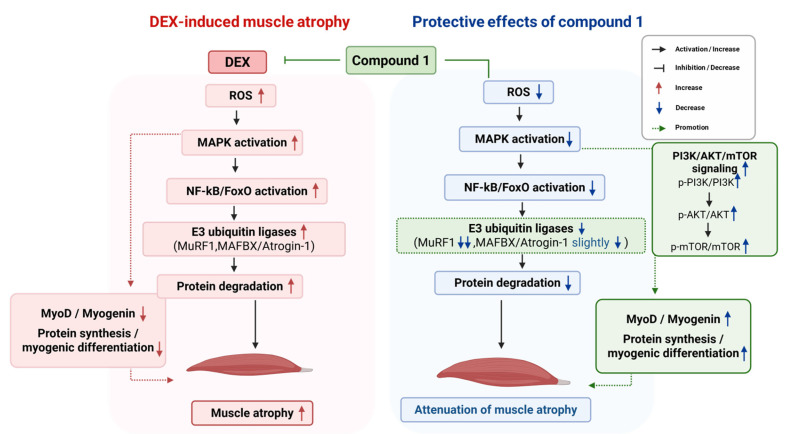
Schematic illustration of the proposed protective effect of compound **1** against DEX-induced muscle atrophy in C2C12 myotubes.

Notably, treatment with compound **1** significantly alleviated these DEX-induced alterations in a concentration-dependent manner. Morphological analysis revealed that compound **1** restored myotube diameter and alignment, indicating a protective effect against structural deterioration. In addition, treatment with compound **1** alone in the absence of DEX slightly increased myotube diameter compared with the untreated control group, suggesting that compound **1** may contribute to the maintenance of myotube integrity under basal conditions. However, further analysis of anabolic signaling markers will be necessary to determine whether this effect reflects direct anabolic activity. Quercetin was included as a reference compound to provide a comparative context for the morphological analysis. The quercetin-treated groups showed recovery of DEX-reduced myotube diameter, providing a useful benchmark for evaluating the relative efficacy of compound **1** in the DEX-induced C2C12 myotube atrophy model. At the molecular level, compound **1** increased the expression of the myogenic regulatory factors MyoD and myogenin and markedly suppressed the atrophy-related E3 ubiquitin ligase MuRF1. In contrast, its suppressive effect on MAFBX/Atrogin-1 was relatively modest, suggesting that compound **1** may preferentially regulate specific components of the ubiquitin–proteasome pathway. In particular, the marked recovery of myogenin expression to levels comparable to or exceeding those of the control group may reflect enhanced myogenic differentiation capacity (Figure 6).

In addition, the present study further showed that compound **1** restored DEX-suppressed PI3K/Akt/mTOR signaling, as indicated by the increased phosphorylation ratios of PI3K, Akt, and mTOR. The PI3K/Akt/mTOR pathway is a major anabolic signaling axis that regulates skeletal muscle protein synthesis, myotube growth, and maintenance. Collectively, these findings suggest that compound **1** may exert its protective effects against DEX-induced muscle atrophy not only by suppressing the expression of atrophy-related proteins, including MuRF1 and MAFBX, but also by restoring PI3K/Akt/mTOR-mediated anabolic signaling in C2C12 myotubes.

Although the precise molecular targets of compound **1** remain unclear, its protective effects may be associated with the modulation of signaling pathways involved in muscle protein turnover. Previous studies have shown that DEX-induced reactive oxygen species (ROS) generation can activate MAPK signaling cascades (JNK, ERK, and p38), which subsequently stimulate NF-κB and FoxO transcription factors [26,27]. These pathways are known to promote the expression of E3 ubiquitin ligases such as MuRF1 and MAFBX/Atrogin-1, thereby accelerating muscle protein degradation [28]. In addition to these catabolic pathways, DEX-induced muscle atrophy is closely associated with suppression of anabolic signaling pathways involved in muscle protein synthesis. In the present study, compound **1** restored the phosphorylation levels of PI3K, Akt, and mTOR, suggesting recovery of PI3K/Akt/mTOR-mediated anabolic signaling in DEX-induced C2C12 myotubes. Together with the suppression of MuRF1 and MAFBX expression, these findings suggest that compound **1** may protect against DEX-induced muscle atrophy by regulating both catabolic protein degradation and anabolic signaling. However, further studies are required to identify the direct molecular targets of compound **1** and to validate the upstream signaling pathways involved in its protective effects. In contrast, compound **4** did not show statistically significant effects on either morphological or molecular markers under the tested conditions, suggesting that its activity may differ in mechanism and warrants further investigation. These findings further imply that structural differences among naphthalene-type glycosides may influence their biological activity, highlighting the potential importance of specific functional groups. Notably, compound **1** contains a malonyl-glucoside moiety, whereas compound **4** differs in both glycosylation pattern and aromatic substitution. Although the present data do not allow definitive conclusions regarding the contribution of malonylation to the observed anti-atrophic activity, the pronounced activity of compound **1** suggests that this structural feature may influence its biological properties. Nevertheless, because only a limited number of structurally related compounds were evaluated, further studies employing additional naphthalene glycosides and synthetic analogs will be necessary to clarify the role of the malonyl-glucoside moiety in mediating biological activity and to establish meaningful structure–activity relationship (SAR) trends.

Taken together, this study demonstrates that compound **1**, a novel naphthalene malonylglucoside isolated from *R. obtusifolius*, exerts protective effects against DEX-induced muscle atrophy by restoring the balance between protein synthesis and degradation. These findings suggest that compound **1** may serve as a promising candidate for the development of functional food ingredients or therapeutic agents targeting muscle atrophy. Despite these promising findings, several limitations should be acknowledged. First, the present study was conducted using an in vitro C2C12 myotube model, which cannot fully reproduce the complex physiological environment of skeletal muscle, including systemic metabolism, hormonal regulation, inflammatory responses, and tissue-level interactions. Therefore, further in vivo studies using animal models of muscle wasting are necessary to validate the protective effects of compound **1** and to evaluate the therapeutic potential of the naphthalene-type glycosides isolated from *R. obtusifolius* under physiologically relevant conditions. Second, although PI3K/Akt/mTOR signaling was investigated in the present study, other pathways known to contribute to skeletal muscle atrophy, including AMPK, ROS/MAPK, NF-κB, and FoxO signaling, were not directly examined. Therefore, additional studies are required to further elucidate the molecular targets and signaling mechanisms underlying the protective effects of compound **1**. Furthermore, future studies incorporating clinically relevant comparators and in vivo efficacy assessments would provide a more comprehensive evaluation of its therapeutic potential.

## 5. Conclusions

Through LC–MS-guided phytochemical investigation of the ethanolic extract of *R. obtusifolius roots*, four naphthalene-type glycosides (**1**–**4**) were isolated and structurally characterized: nepodin-8-*O*-*β*-d-(6′-*O*-malonyl)-glucopyranoside (**1**), torachrysone-8-*O*-*β*-D-(6′-*O*-malonyl)-glucopyranoside (**2**), nepodin-8-*O*-*β*-D-glucopyranoside (**3**), and torachrysone-8-*O*-*β*-D-glucopyranoside (**4**). Among these, compound **1** was identified as a new compound, and its structure was elucidated using LC–MS and NMR spectroscopic analyses, supported by chemical transformation. Furthermore, the effects of the isolated compounds **1** and **4** were evaluated on DEX-induced muscle atrophy in C2C12 myotubes. Compound **1** significantly attenuated DEX-induced muscle atrophy in a concentration-dependent manner, as evidenced by the increased myotube diameter and improved morphological features. In contrast, compound **4** did not show statistically significant effects under the tested conditions. Compound **1** also restored the expression of myogenic markers (MyoD and myogenin), suppressed the expression of atrophy-related proteins (MuRF1 and MAFBX/Atrogin-1), and reversed the DEX-induced suppression of PI3K/Akt/mTOR signaling, suggesting modulation of both anabolic and catabolic pathways involved in muscle homeostasis. Overall, these findings highlight compound **1** as a promising lead compound with significant therapeutic potential for the prevention and treatment of muscle atrophy.

## Figures and Tables

**Table 1:** ^1^H (850 MHz) and ^13^C NMR (212.5 MHz) data of compounds **1** and **2** in DMSO-*d*_6_ (*δ* ppm) *^a^*.

Position	1		2	
	*δ*_C_, Type	*δ*_H_ (*J* in Hz)	*δ*_C_, Type	*δ*_H_ (*J* in Hz)
1	150.6, C		150.9, C	
2	125.3, C		123.3, C	
3	133.7, C		133.7, C	
4	119.3, CH	7.22, s	118.9, CH	7.10, s
5	122.5, CH	7.48, d (8.0)	101.1, CH	6.91, d (2.0)
6	127.4, CH	7.44, t (8.0)	158.2, C	
7	110.6, CH	7.26, d (8.0)	103.6, CH	6.95, d (2.0)
8	154.4, C		155.1, C	
9	113.3, C		108.6, C	
10	136.1, C		136.9, C	
11	205.0, C		204.5, C	
12	32.0, CH_3_	2.53, s	32.2, CH_3_	2.51, s
13	19.5, CH_3_	2.25, s	19.5, CH_3_	2.23, s
1′	102.2, CH	5.09, d (8.0)	102.3, CH	5.10, d (8.0)
2′	73.1, CH	3.40, m	73.2, CH	3.37, d (6.5)
3′	75.1, CH	3.37, m	76.0, CH	3.35, m
4′	69.8, CH	3.24, m	70.0, CH	3.24, m
5′	74.2, CH	3.72, ddd (9.5, 7.5, 2.0)	74.3, CH	3.75, m
6′	64.1, CH_2_	4.16, dd (12.0, 7.5); 4.48, dd (12.0, 2.0)	63.9, CH_2_	4.14, dd (11.0, 7.5); 4.42, d (11.0)
6-OCH_3_			55.4, CH_3_	3.83, s
1″	167.6, C		167.4, C	
2″	41.7, CH_2_	3.42, m	42.1, CH_2_	3.39, m
3″	168.2, C		168.2, C	

*^a^* Coupling constants (Hz) are given in parentheses; ^13^C NMR data were assigned based on the HSQC and HMBC experiments.

## Data Availability

The data supporting this study’s findings are available from the corresponding author upon reasonable request.

## Supplementary Materials

The following supporting information can be downloaded at: https://www.mdpi.com/article/10.3390/pharmaceutics18070807/s1, Figure S1: The HR-ESI-MS data of **1**; Figure S2: The UV spectrum of **1**; Figure S3: The ^1^H NMR spectrum of **1** (DMSO-*d*_6_, 850 MHz); Figure S4: The ^1^H−^1^H COSY spectrum of **1**; Figure S5: The HSQC spectrum of **1**; Figure S6: The HMBC spectrum of **1**; Figure S7: The HR-ESI-MS data of **2**; Figure S8: The UV spectrum of **2**; Figure S9: The ^1^H NMR spectrum of **2** (DMSO-*d*_6_, 850 MHz); Figure S10: The ^13^C NMR spectrum of **2** (DMSO-*d*_6_, 212.5 MHz); Figure S11: The HR-ESI-MS data of **3**; Figure S12: The UV spectrum of **3**; Figure S13: The ^1^H NMR spectrum of **3** (CD3OD, 850 MHz); Figure S14: The HR-ESI-MS data of **4**; Figure S15: The UV spectrum of **4**; Figure S16: The ^1^H NMR spectrum of **4** (acetone-*d*_6_, 850 MHz).
