# Supplementary material for: Naphthalene-Type Glycosides from Rumex obtusifolius Roots and Their Protective Effects Against Muscle Atrophy in C2C12 Myotubes
Source: Pharmaceutics. 2026 Jun 29;18(7):807. doi: 10.3390/pharmaceutics18070807 (PMC13414561; doi:10.3390/pharmaceutics18070807)

Supplementary Materials

Naphthalene-type Glycosides from *Rumex obtusifolius* Roots and their Protective Effects against Muscle Atrophy in C2C12 Myotubes

Yun Seok Joh [1,†], Jung Eun Park [2,†], Moon Jin Ra [3], Sang Mi Jung [3], Gabsik Yang [2], Ki Sung Kang [2,*], Ki Hyun Kim [1,*]

[1]School of Pharmacy, Sungkyunkwan University, Suwon 16419, Republic of Korea; ysjoh05@g.skku.edu (Y.S.J.)
[2]College of Korean Medicine, Gachon University, Seongnam 13120, Republic of Korea; ppp1416@gachon.ac.kr (J.E.P.), yanggs@gachon.ac.kr (G.Y.)
[3]Hongcheon Institute of Medicinal Herb, Hongcheon-gun, Gangwon-do 25142, Republic of Korea; ramj90@himh.re.kr (M.J.R.); sgmo77@naver.com (S.M.J.)
[*]Correspondence: kkang@gachon.ac.kr (K.S.K.); khkim83@skku.edu (K.H.K.); Tel.: +82-31-290-7700 (K.H.K.)
[†]These authors contributed equally to this study.

Supporting Information Contents:

Figure S1. The HR-ESI-MS data of **1** S3
Figure S2. The UV spectrum of **1** S4
Figure S3. The ^{1}H NMR spectrum of **1** (DMSO-d_6, 850 MHz) S5
Figure S4. The ^{1}H-^{1}H COSY spectrum of **1** S6
Figure S5. The HSQC spectrum of **1** S7
Figure S6. The HMBC spectrum of **1** S8
Figure S7. The HR-ESI-MS data of **2** S9
Figure S8. The UV spectrum of **2** S10
Figure S9. The ^{1}H NMR spectrum of **2** (DMSO-d_6, 850 MHz) S11
Figure S10. The ^{13}C NMR spectrum of **2** (DMSO-d_6, 212.5 MHz) S12
Figure S11. The HR-ESI-MS data of **3** S13
Figure S12. The UV spectrum of **3** S14
Figure S13. The ^{1}H NMR spectrum of **3** (CD_3OD, 850 MHz) S15
Figure S14. The HR-ESI-MS data of **4** S16
Figure S15. The UV spectrum of **4** S17
Figure S16. The ^{1}H NMR spectrum of **4** (acetone-d_6, 850 MHz) S18

Figure S1. The HR-ESIMS data of **1**

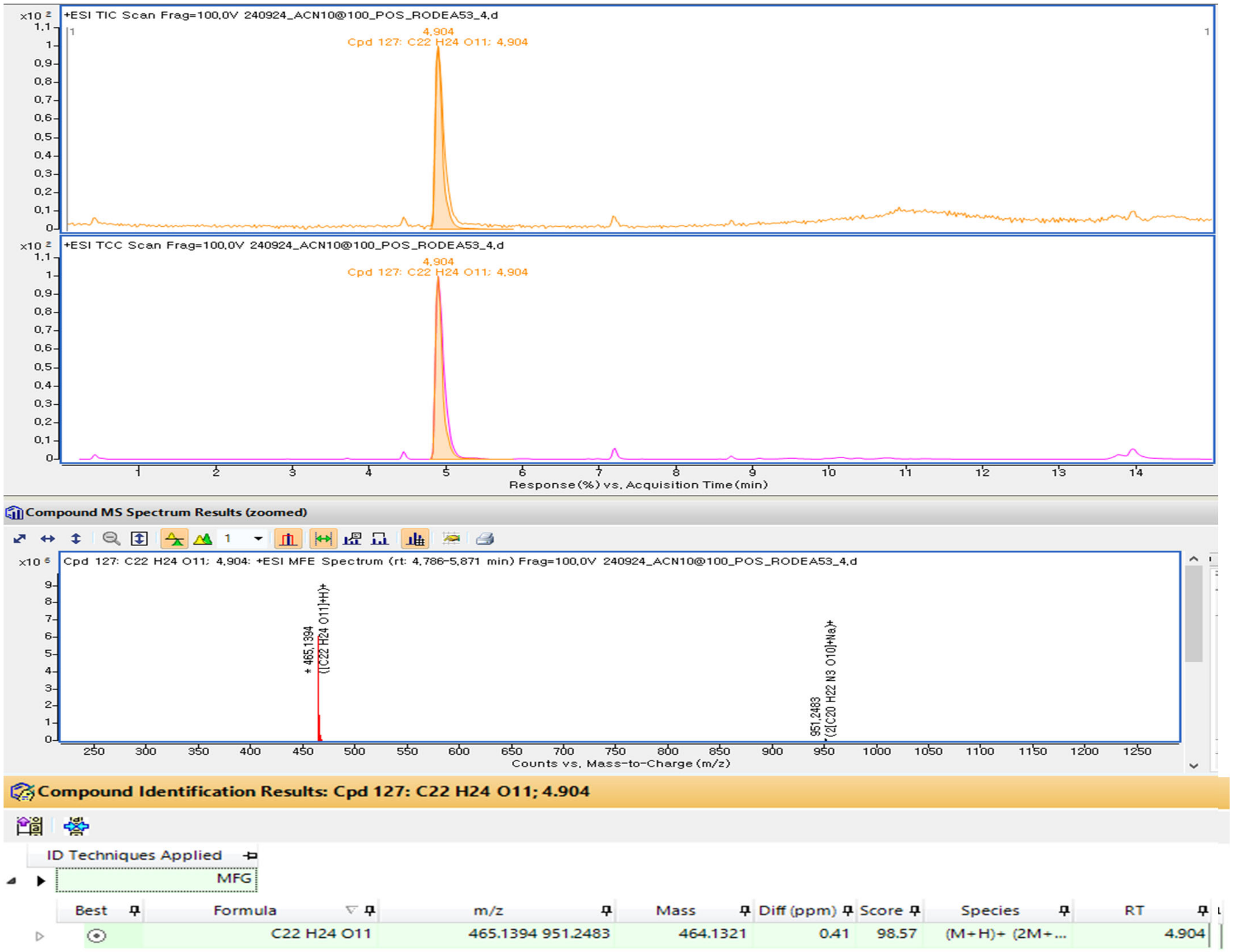

Figure S2. The UV spectrum of **1**

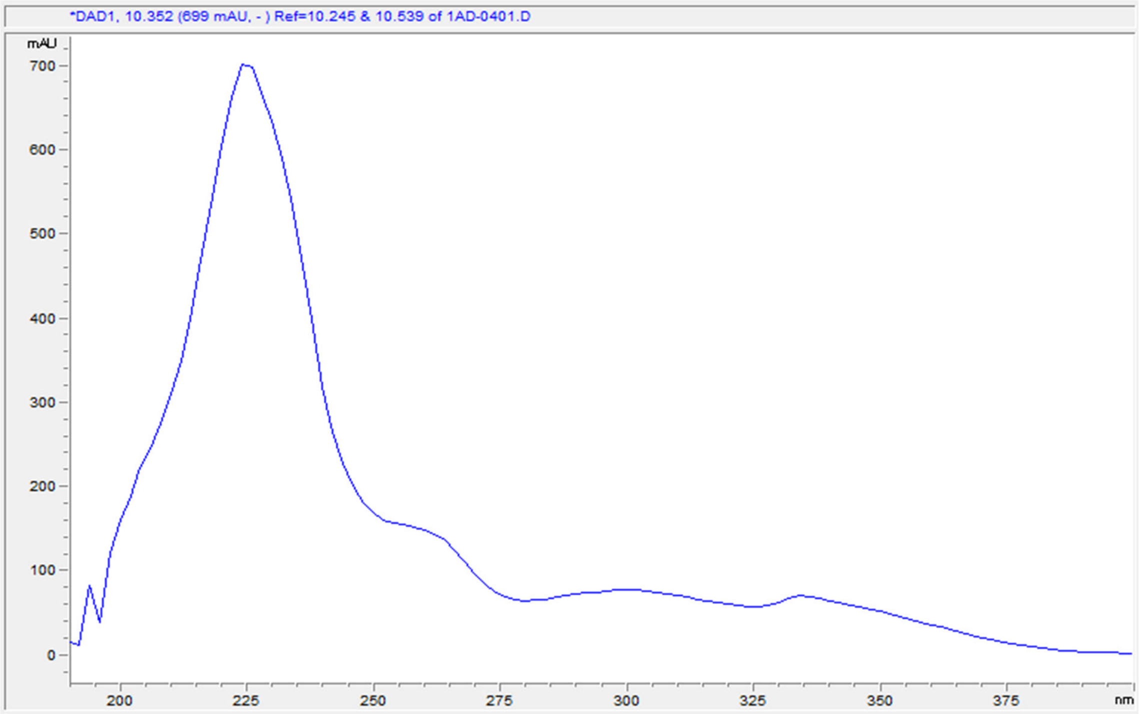

Figure S3. The ^{1}H NMR spectrum of **1** (DMSO-d_6, 850 MHz)

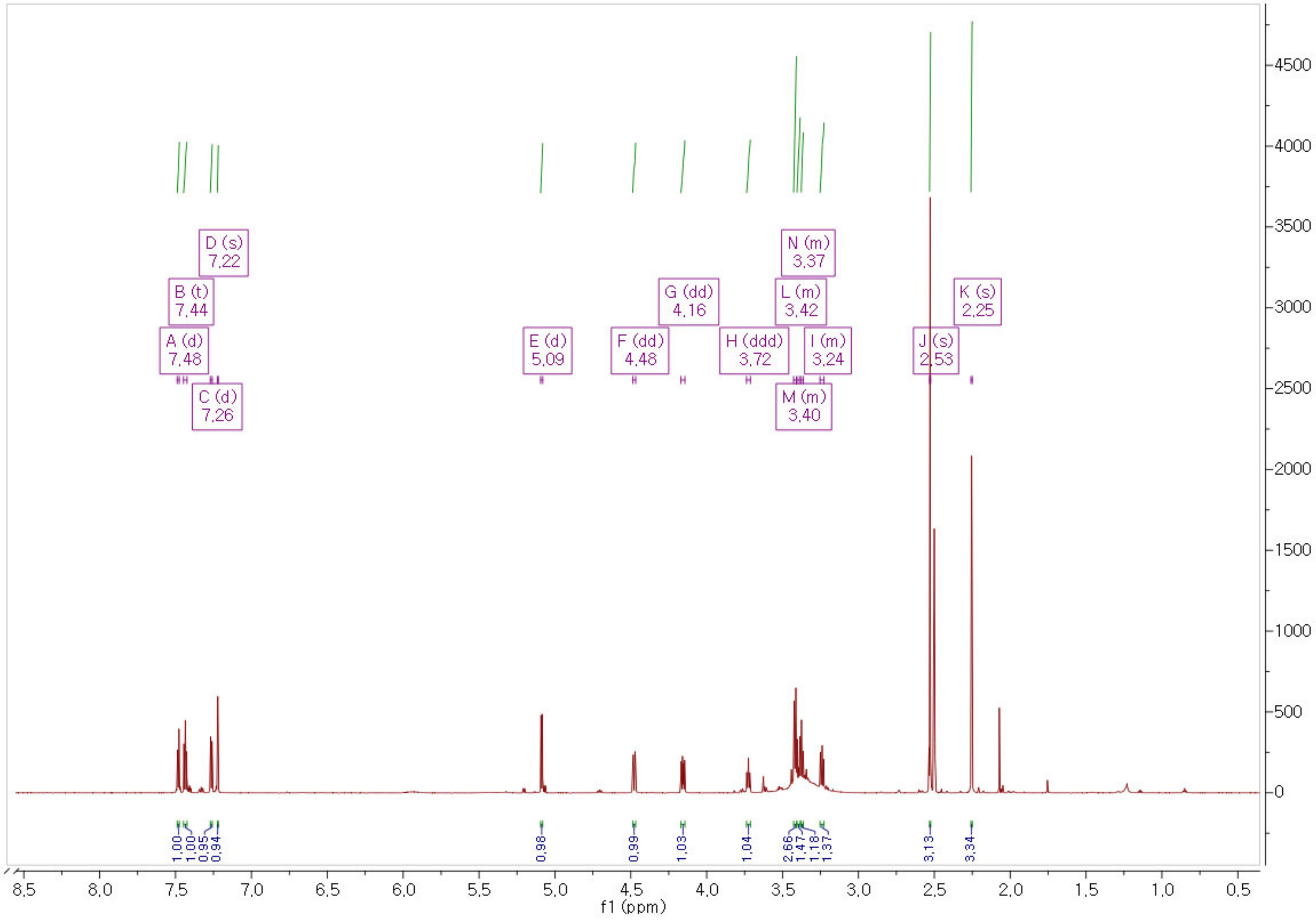

Figure S4. The ^{1}H-^{1}H COSY spectrum of **1**

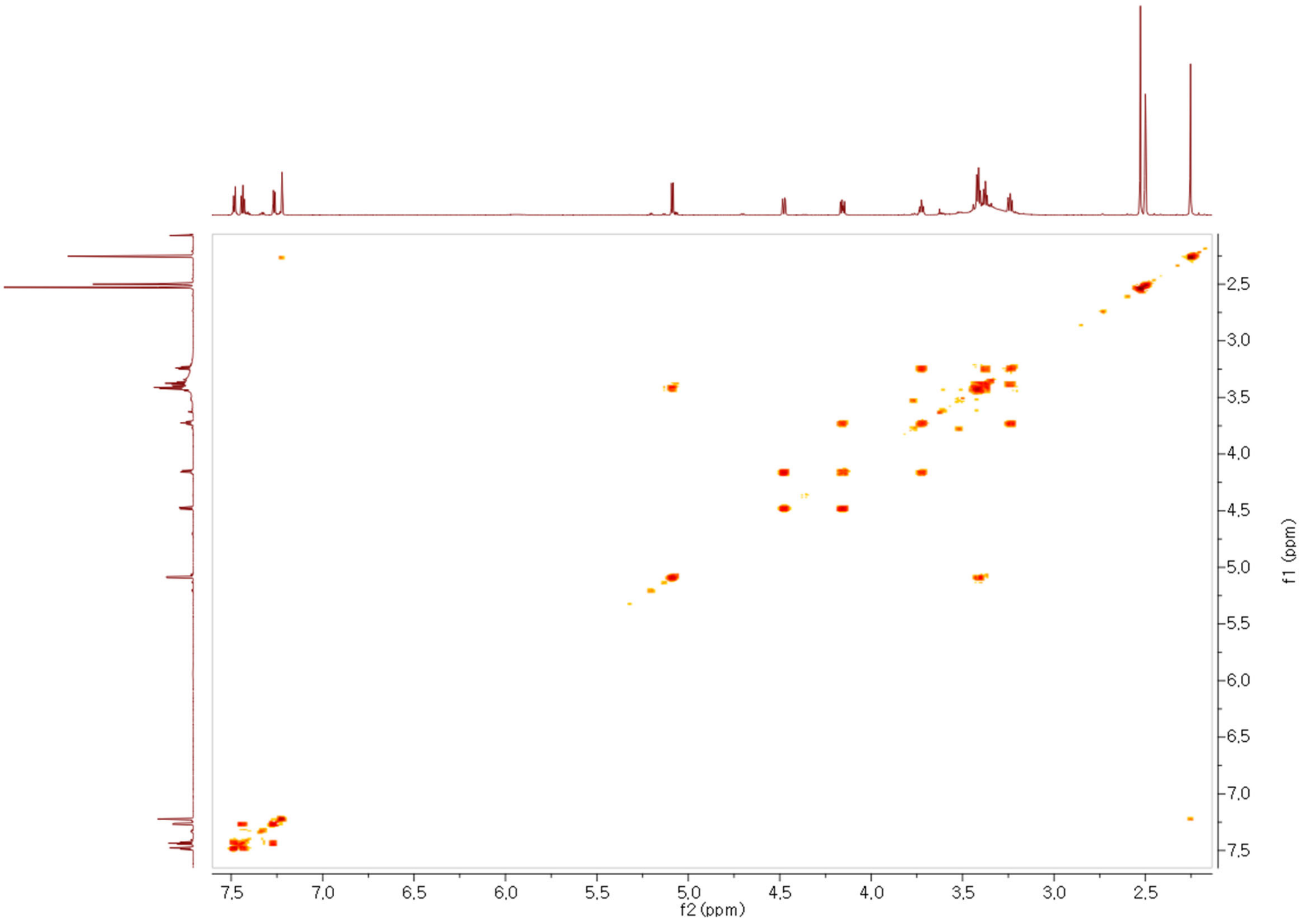

Figure S5. The HSQC spectrum of **1**

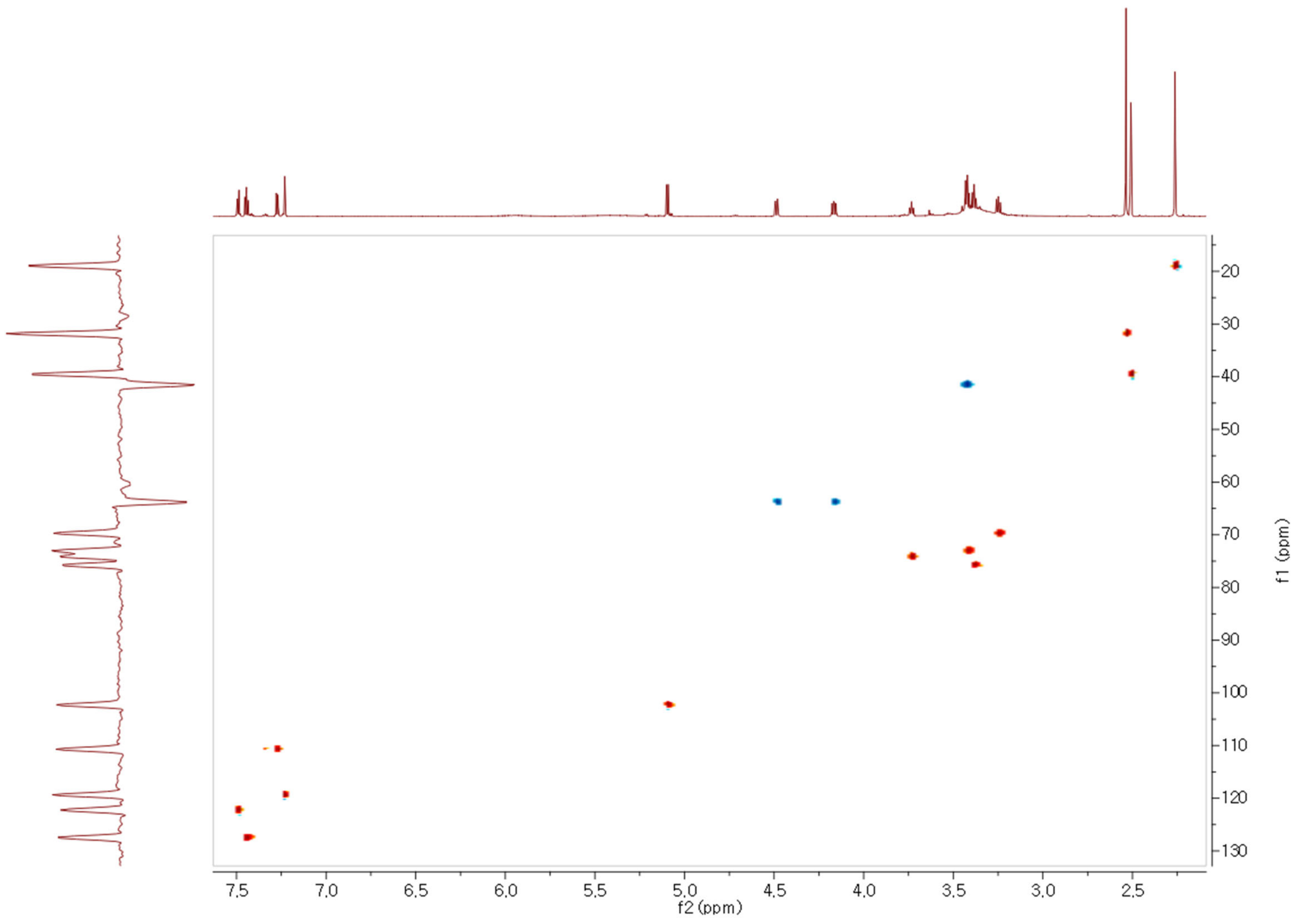

Figure S6. The HMBC spectrum of **1**

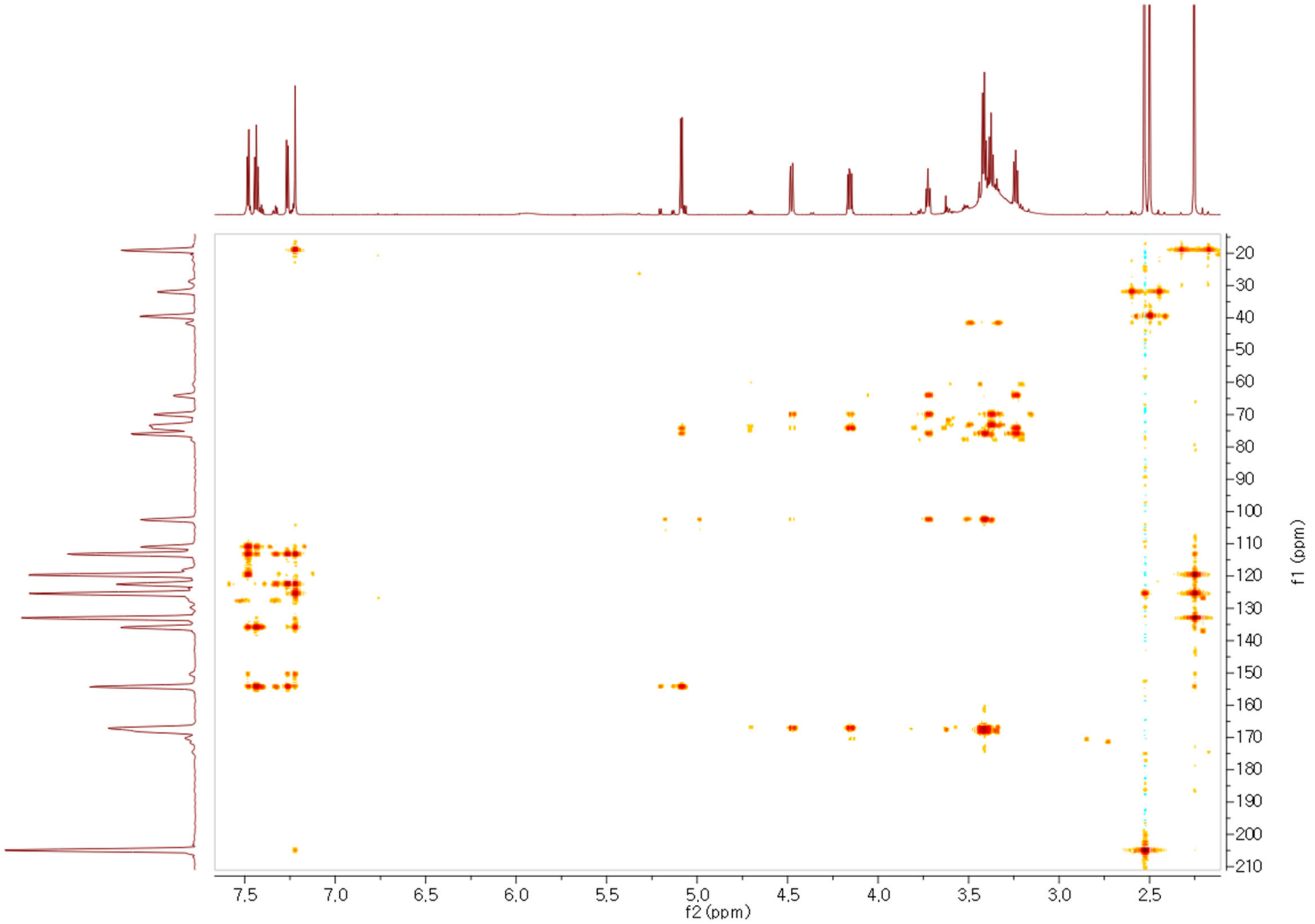

Figure S7. The HR-ESIMS data of **2**

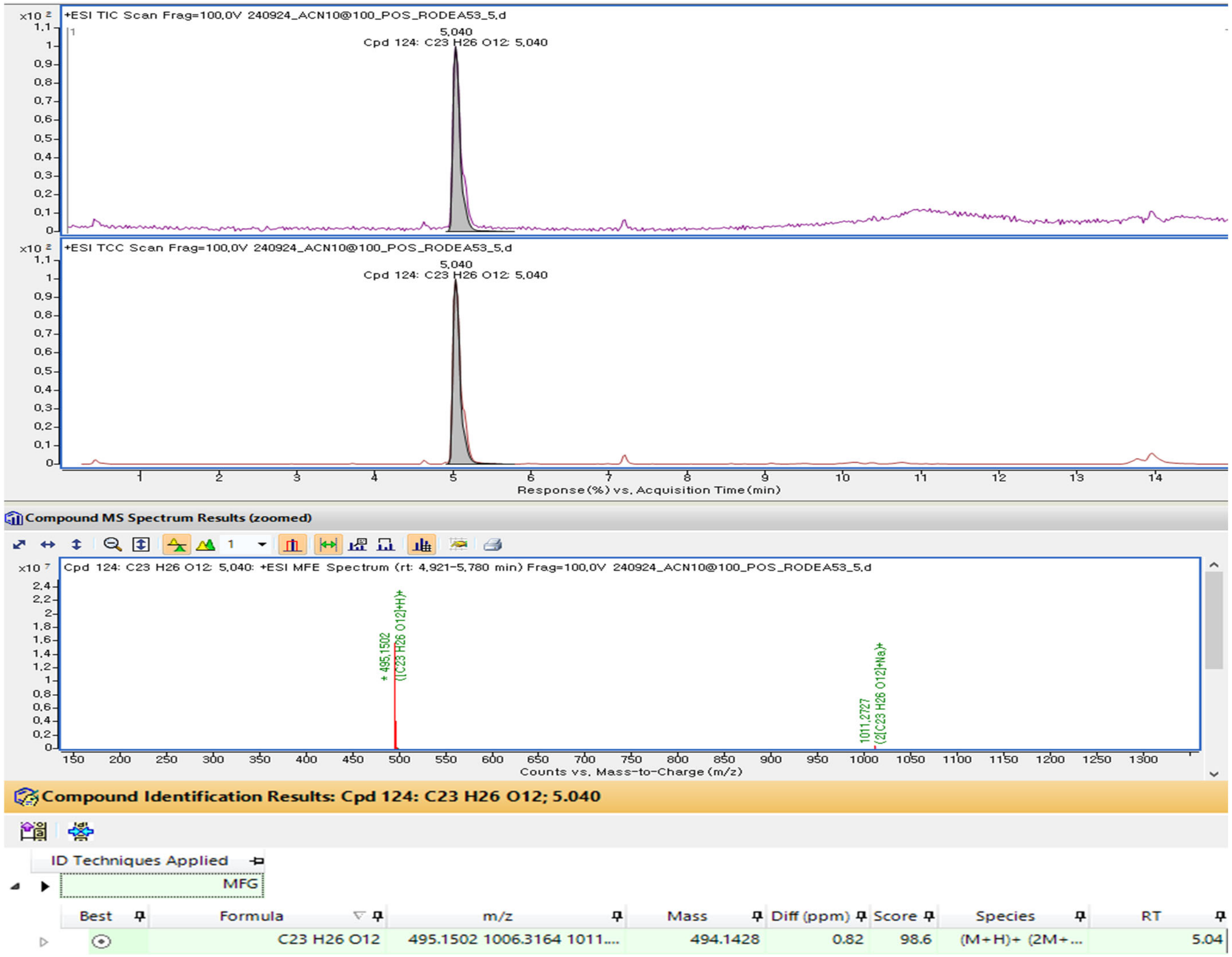

Figure S8. The UV spectrum of **2**

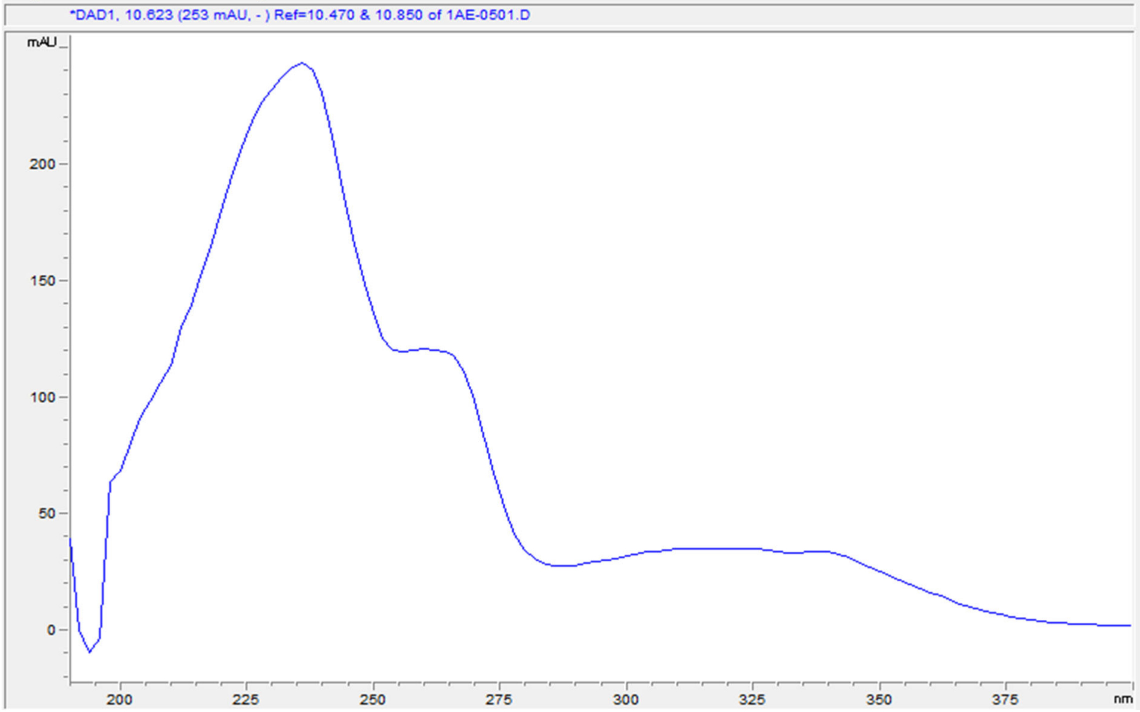

Figure S9. The ^{1}H NMR spectrum of **2** (DMSO-d_6, 850 MHz)

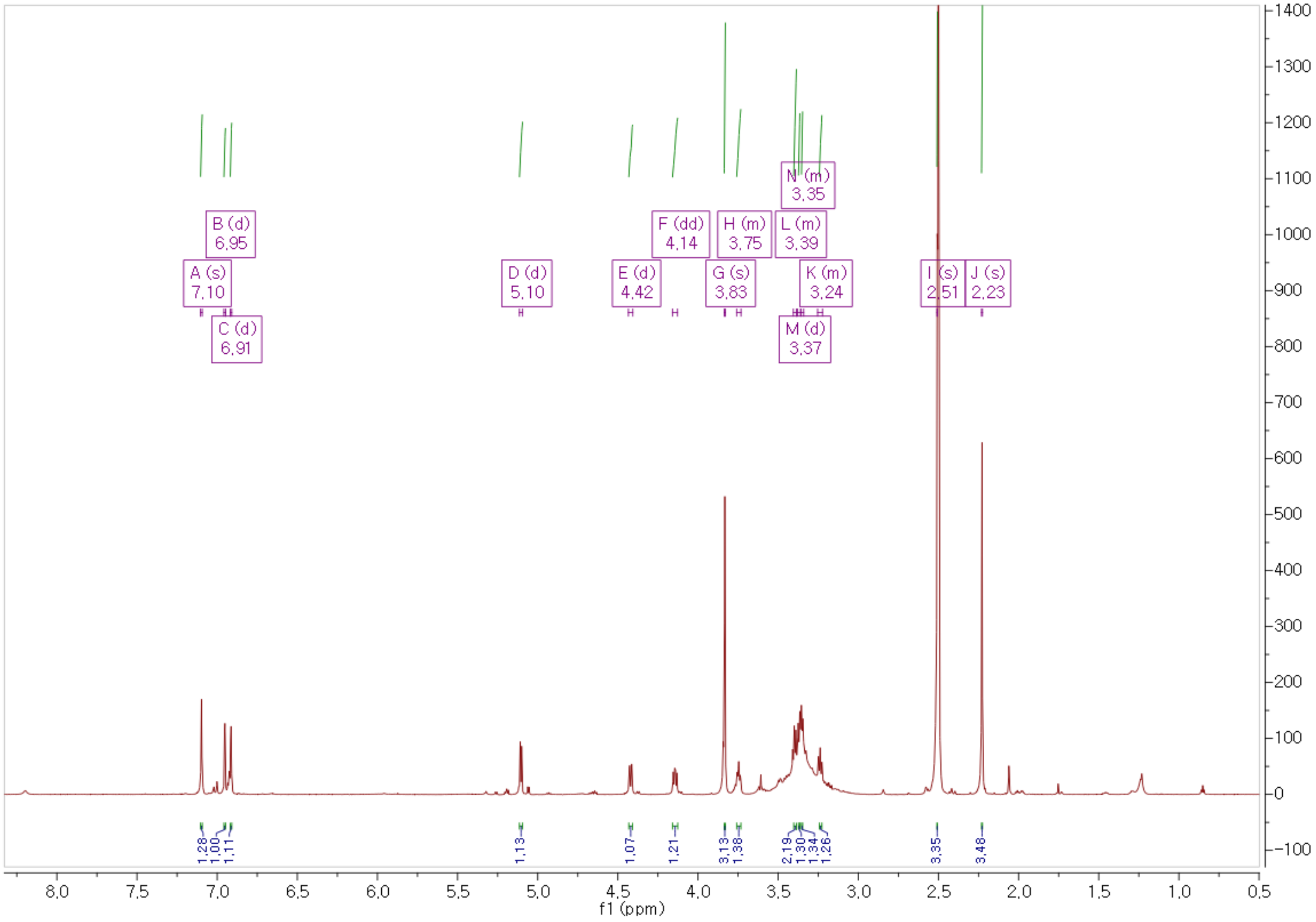

Figure S10. The ^{13}C NMR spectrum of **2**

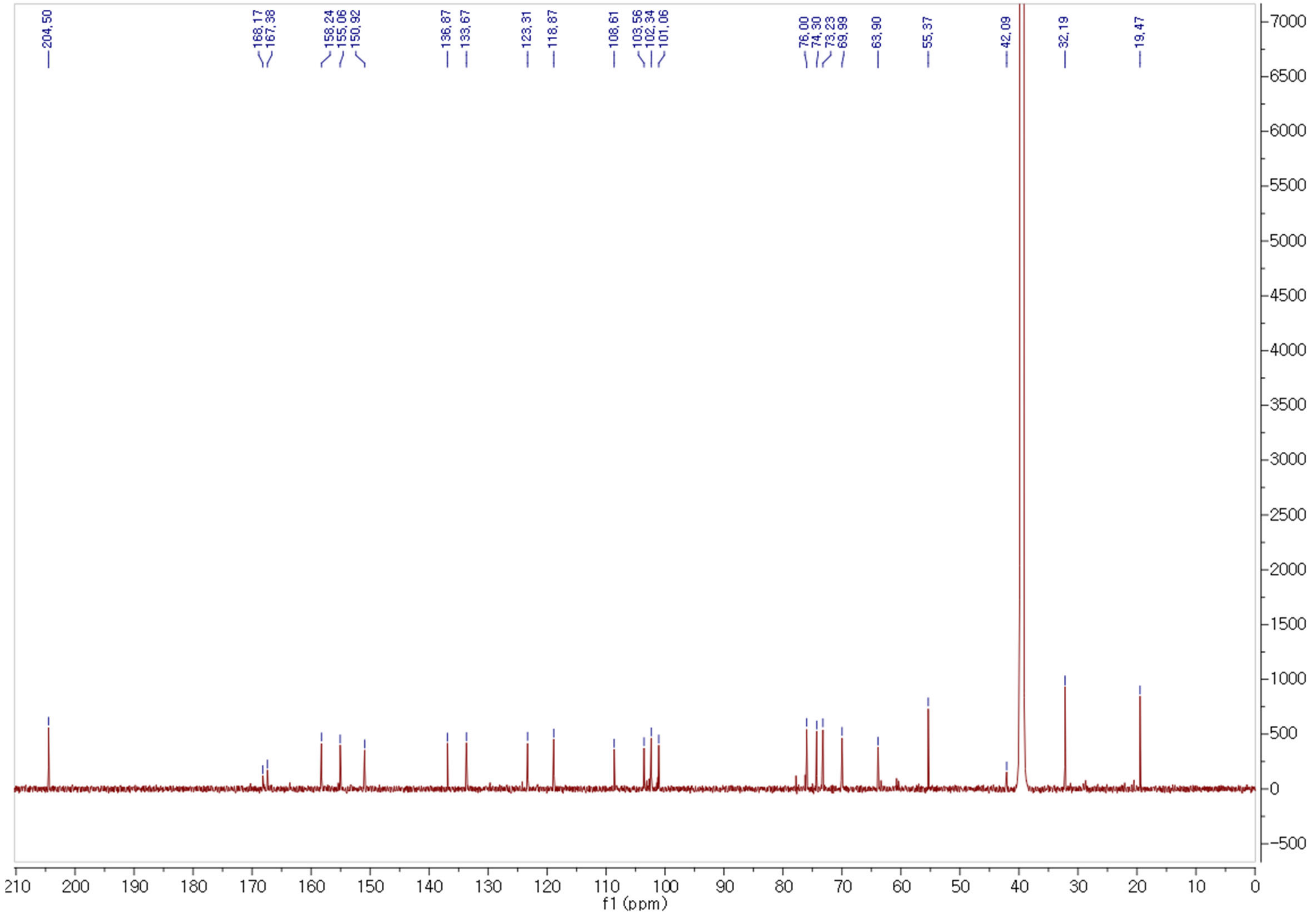

Figure S11. The HR-ESIMS data of **3**

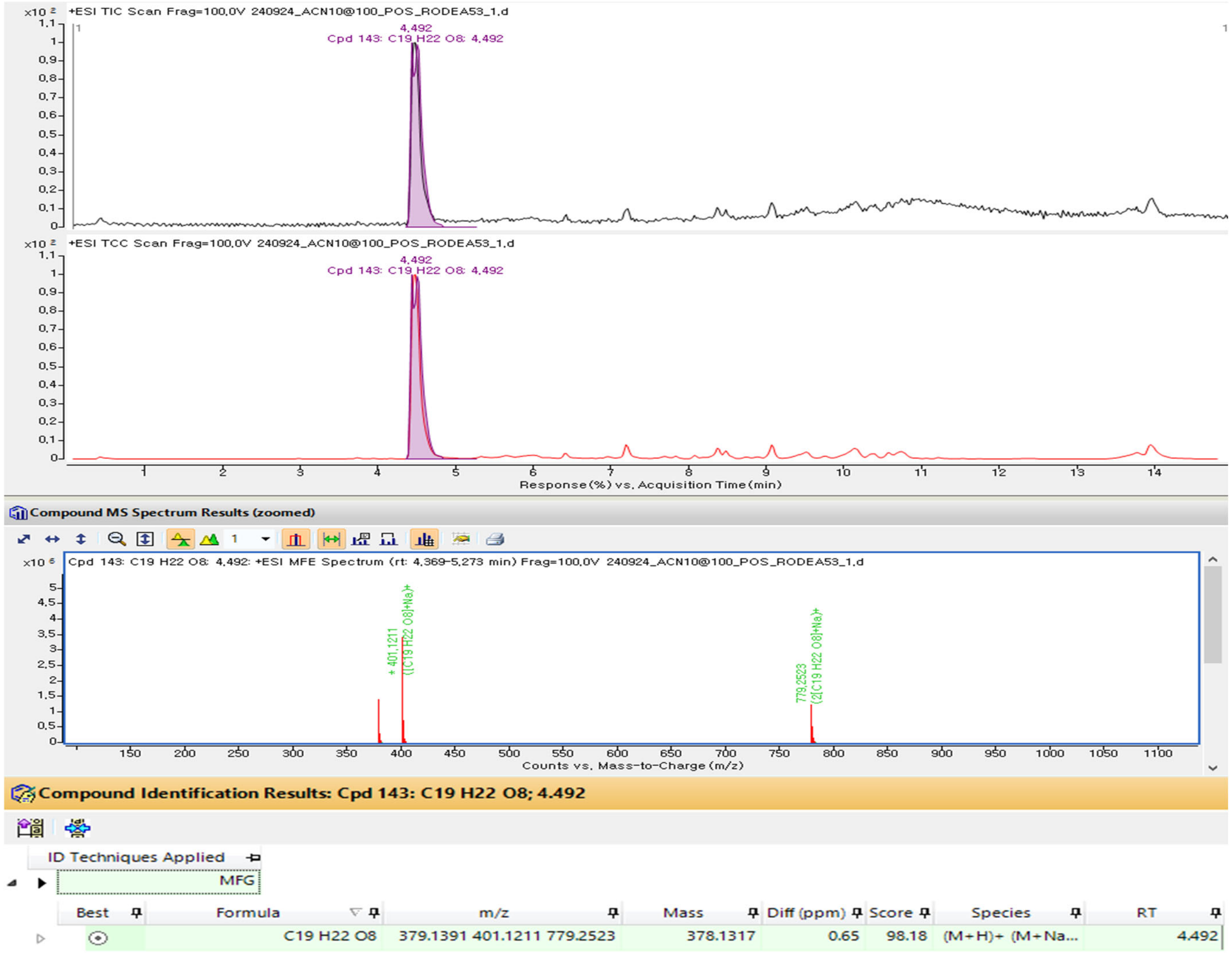

Figure S12. The UV spectrum of **3**

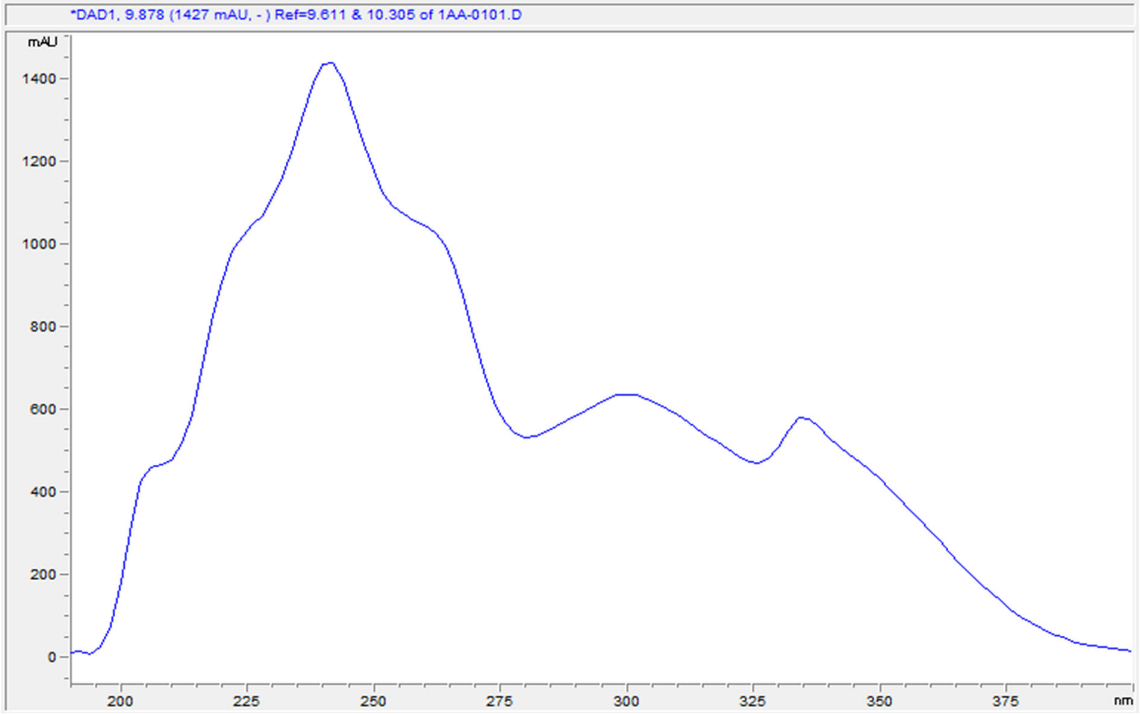

Figure S13. The ^{1}H NMR spectrum of **3** (CD_3OD, 850 MHz)

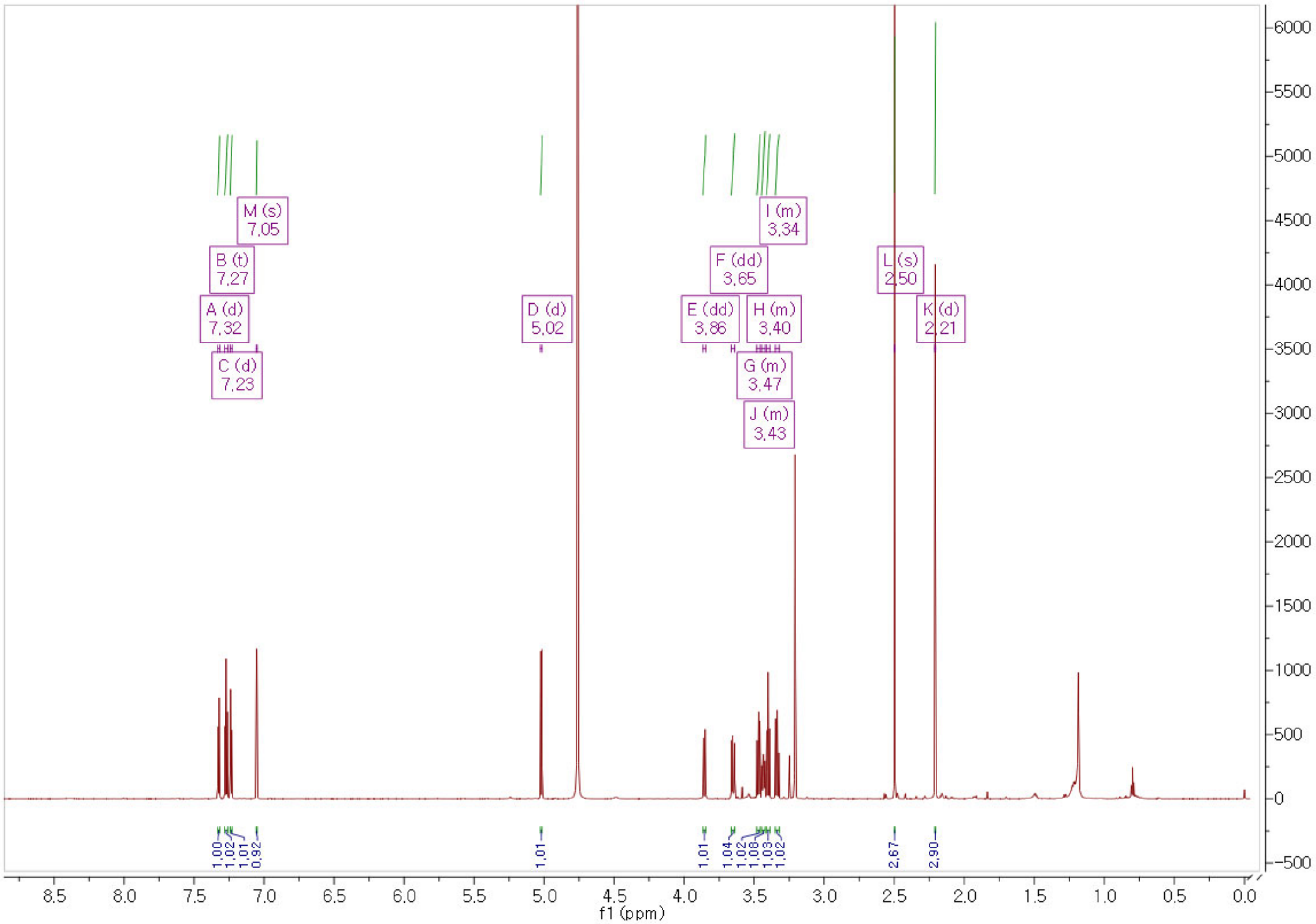

Figure S14. The HR-ESIMS data of **4**

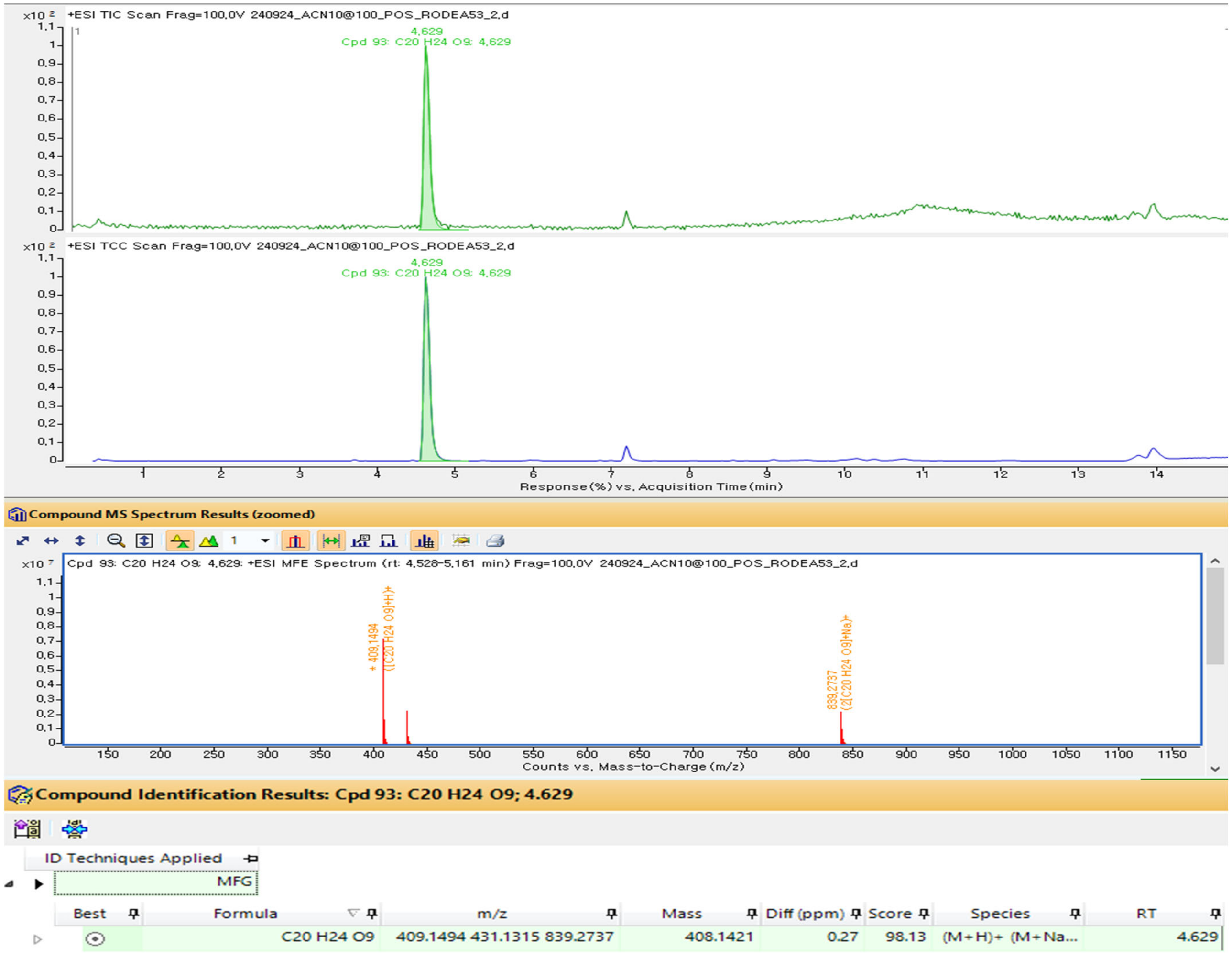

Figure S15. The UV spectrum of **4**

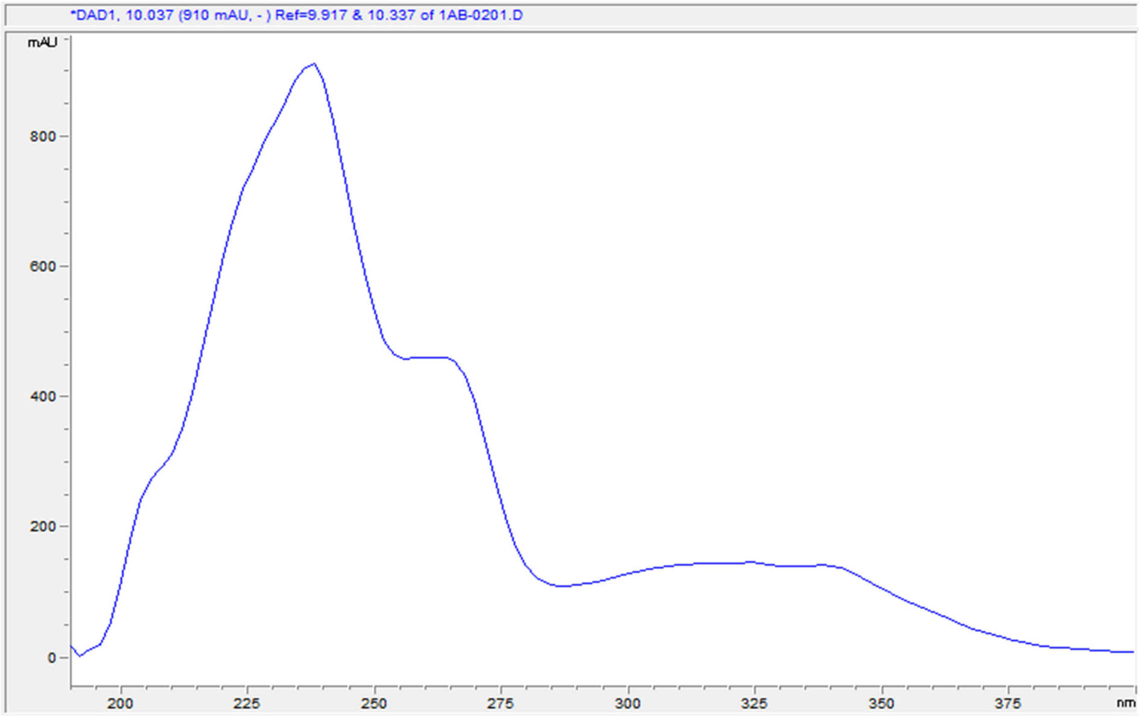

Figure S16. The ^{1}H NMR spectrum of **3** (acetone-d_6, 850 MHz)

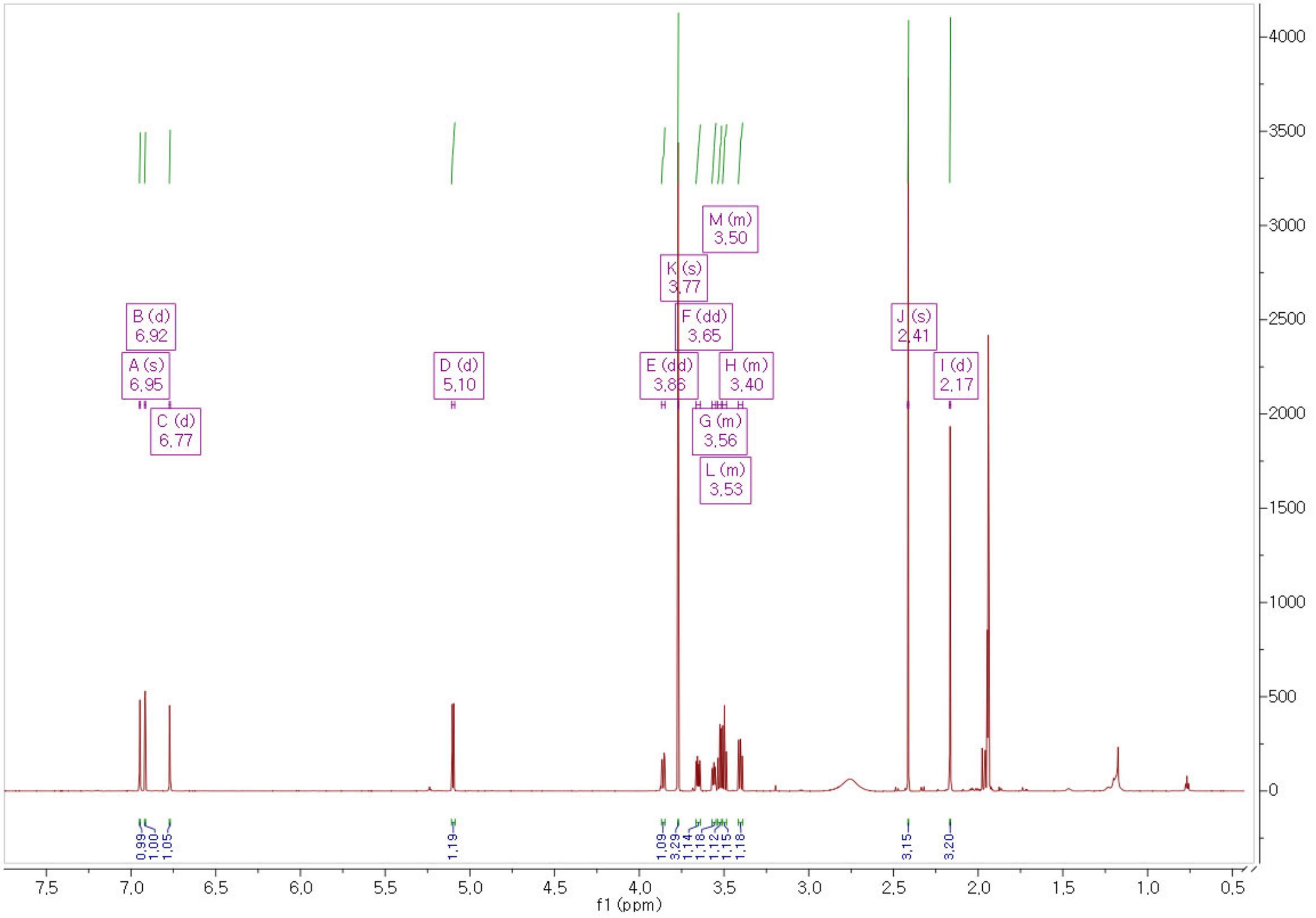

Supplement: Supplementary file 1 [file pharmaceutics-18-00807-s001.zip › pharmaceutics-4318980-supplementary.pdf]
